# ssMutPA: single-sample mutation-based pathway analysis approach for cancer precision medicine

**DOI:** 10.1093/gigascience/giae105

**Published:** 2024-12-20

**Authors:** Yalan He, Jiyin Lai, Qian Wang, Bingyue Pan, Siyuan Li, Xilong Zhao, Ziyi Wang, Yongbao Zhang, Yujie Tang, Junwei Han

**Affiliations:** College of Bioinformatics Science and Technology, Harbin Medical University, Harbin 150081, China; College of Bioinformatics Science and Technology, Harbin Medical University, Harbin 150081, China; College of Bioinformatics Science and Technology, Harbin Medical University, Harbin 150081, China; College of Bioinformatics Science and Technology, Harbin Medical University, Harbin 150081, China; College of Bioinformatics Science and Technology, Harbin Medical University, Harbin 150081, China; College of Bioinformatics Science and Technology, Harbin Medical University, Harbin 150081, China; College of Bioinformatics Science and Technology, Harbin Medical University, Harbin 150081, China; College of Bioinformatics Science and Technology, Harbin Medical University, Harbin 150081, China; College of Bioinformatics Science and Technology, Harbin Medical University, Harbin 150081, China; College of Bioinformatics Science and Technology, Harbin Medical University, Harbin 150081, China

**Keywords:** somatic mutation, network topology, single-sample pathway analysis, cancer subtypes, precision medicine

## Abstract

**Background:**

Single-sample pathway enrichment analysis is an effective approach for identifying cancer subtypes and pathway biomarkers, facilitating the development of precision medicine. However, the existing approaches focused on investigating the changes in gene expression levels but neglected somatic mutations, which play a crucial role in cancer development.

**Findings:**

In this study, we proposed a novel single-sample mutation-based pathway analysis approach (ssMutPA) to infer individualized pathway activities by integrating somatic mutation data and the protein–protein interaction network. For each sample, ssMutPA first uses local and global weighted strategies to evaluate the effects of genes from mutations according to the network topology and then calculates a single-sample mutation-based pathway enrichment score (ssMutPES) to reflect the accumulated effect of mutations of each pathway. To illustrate the performance of ssMutPA, we applied it to 33 cancer cohorts from The Cancer Genome Atlas database and revealed patient stratification with significantly different prognosis in each cancer type based on the ssMutPES profiles. We also found that the identified characteristic pathways with high overlap across different cancers could be used as potential prognosis biomarkers. Moreover, we applied ssMutPA to 2 melanoma cohorts with immunotherapy and identified a subgroup of patients who may benefit from therapy.

**Conclusions:**

We provided evidence that ssMutPA could infer mutation-based individualized pathway activity profiles and complement the current individualized pathway analysis approaches focused on gene expression data, which may offer the potential for the development of precision medicine. ssMutPA is available at https://CRAN.R-project.org/package=ssMutPA.

## Graphical Abstract

**Graphical Abstract:**
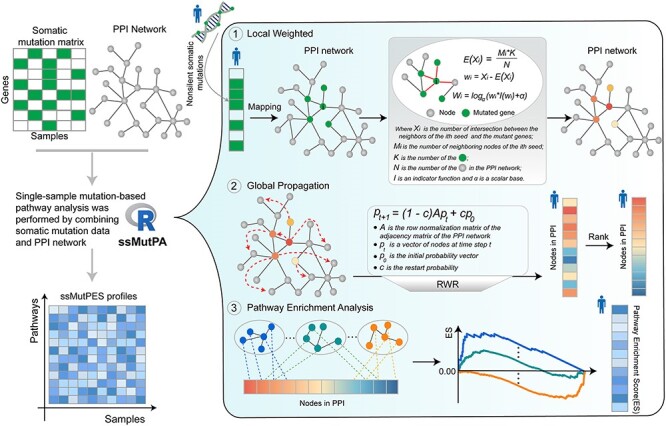

## Background

Over the past decades, gene signatures derived from transcriptomics data have been recognized in multiple cancers [1–5]. However, their clinical application has been hindered by low reproducibility and small overlap [6–8]. Recently, many studies have proved that cancers are essentially caused by disturbances in the complex regulatory relationships among multiple functional genes, suggesting the need to convert gene expression data into pathway-level activity values for further studies [9, 10].

Pathway enrichment analysis (PEA) is currently the most popular method to interpret transcriptomics data using knowledge of gene sets or biological pathways. Thus, more and more PEA algorithms have been proposed for identifying biomarkers and cancer subtypes, such as gene set enrichment analysis (GSEA) [11], signaling pathway impact analysis (SPIA) [12], and CTpathway [13]. While these methods converted traditional gene expression data into pathway-level analysis and explored the dysregulated pathways between the 2 phenotypes, their effectiveness relied on a large number of sample data. More importantly, they ignore individualized patient information on pathways. To overcome these limitations, individualized pathway activity calculation methods and tools have been evolved, such as single-sample GSEA (ssGSEA) [14], gene set variation analysis (GSVA) [15], iPath [16], Pathifier [17], and so on. ssGSEA was introduced by Barbie et al. [14], who extended the GSEA algorithm to the single-sample level and calculated the enrichment statistic for each pathway on a single sample, thus reflecting the extent to which the genes contained in a particular pathway are up- or downregulated in each sample. Similarly, GSVA estimated changes in pathway activity on a sample via an unsupervised manner, which first calculated the expression statistics of the kernel estimates on a sample and subsequently figured up the activity of each pathway in a single sample, providing greater power to detect subtle changes in pathway activity across sample populations. Su et al. [16] developed the iPath algorithm to classify tumor samples into 2 distinct groups by calculating pathway-based individual-level enrichment scores. The algorithm was applied in the pan-cancer analysis, and the results showed that iPath could effectively identify the pathway markers associated with overall survival, subtype, and stage of cancers. In Pathifier, for a tumor sample, a dysregulation score was assigned to each pathway by estimating the extent to which the pathway in that sample deviated from the normal samples, thereby reflecting the pathway’s activity. All the methods described above rely on gene expression data, which is quite dynamic due to batch effects and many other factors [18, 19]. These problems would contribute to some extent to the low reliability and poor clinical applicability of the markers identified by these methods. Therefore, there is an urgent need to develop more comprehensive methods for individualized pathway analysis using other omics data.

Modern medicine has proved that the accumulation of genetic mutations is an important cause of cancers and plays an essential role in the occurrence and development of cancers [20–23]. In clinical practice, the mutation data were used far more commonly than other omics analyses. Although many mutation-based biomarkers have been identified in recent studies [24–27], they are difficult to consider the combined effects of the mutated genes in pathways for a single sample because of the sparseness and discreteness of mutation data. Therefore, the development of mutation-based individualized pathway analysis is urgently needed to identify cancer subtypes and biomarkers for precise treatment of patients.

Here, we innovatively proposed an approach called single-sample mutation-based pathway analysis (ssMutPA), which integrated somatic mutation data and the protein–protein interaction (PPI) network topology to infer individualized pathway activity profiles induced by mutations. ssMutPA considered the positions of mutation genes in the PPI network for each sample and used local and global weighted strategies to evaluate the potential influence extent of genes from mutations. Then, it calculated the mutation-based pathway enrichment scores to reflect the individualized pathway activities induced by mutations. This will complement the current individualized pathway analysis approaches’ focus on gene expression data and provide something new insight into the initiation and progression of cancer. We applied ssMutPA to 33 cancers from The Cancer Genome Atlas (TCGA) database and identified cancer subtypes with significant prognostic differences in 32 cancer types. The identified coherent pathways across these cancers could be used as effective prognostic biomarkers. In addition, by applying ssMutPA to 2 melanoma cohorts treated with immune checkpoint inhibitors (ICIs), the patients were classified into 2 subgroups with significantly different immunotherapy responses based on pathway activity profiles. Ultimately, to facilitate the use of our method, ssMutPA was developed as an R-based package, which is freely available on the Comprehensive R Archive Network (CRAN) [28].

## Materials and Methods

### Data collection and processing

To demonstrate the effectiveness and applicability of ssMutPA, we downloaded somatic mutation data and corresponding clinical information for 33 cancer types from the TCGA database [29]. In this study, we focused exclusively on nonsilent mutations extracted from Mutation Annotation Format (MAF) files. For each cancer type, we used primary tumor samples for our analysis. In addition, we obtained 2 independent glioma datasets (Varn et al. cohort and Wang et al. cohort) from the cBioPortal [30] to validate the generalizability and robustness of the ssMutPA method [31, 32]. To further explore whether our approach can be applied to patients treated with ICIs, we collected 2 datasets treated with ICIs from the cBioPortal database [30] and published literature [33, 34]. The Liu et al. dataset contains somatic mutation data and clinical information (overall survival, response to immunotherapy, etc.) for 105 patients with melanoma with a cutaneous primary and who were treated with programmed cell death protein 1 (PD-1) blockade. In this dataset, response to tumor immunotherapy was defined according to the Response Evaluation Criteria in Solid Tumors 1.1 (RECIST 1.1) criteria. Patients with complete response (CR) or partial response (PR) were considered responders; patients with stable disease (SD) or progressive disease (PD) were considered nonresponders. Another dataset curated by Snyder et al. included 44 patients with primary cutaneous melanoma who received T-lymphocyte-associated antigen 4 (CTLA-4) blockade therapies. Unlike the dataset presented by Liu et al., this cohort defined patients with long benefit (LB) as responders and patients with nonbenefit (NB) as nonresponders. Detailed information on all cohorts used in this study is provided in Supplementary Tables S1–S3.

We downloaded 323 pathways from the KEGG database [35, 36], encompassing metabolism, membrane transport, signal transduction, cell cycle, and so on. The human-specific PPI network was obtained from 12 sources collected by previous researchers [37, 38]. To obtain high-confidence links, we further filtered PPIs from more than 2 sources. This measure for filtering PPIs has been used in previous studies [13]. Finally, the largest connected subnetwork containing 12,436 nodes and 83,020 edges was extracted using the “igraph” package. Compared to directly using KEGG pathways for network construction (323 pathways with 5,954 genes), the PPI network encompasses a more comprehensive human genes.

### The ssMutPA framework

ssMutPA was developed to calculate the mutation-based individualized pathway activity profiles. To do this, we mapped the somatic mutations to the genes in the PPI network as seed nodes for each individual sample. Then, the local and global weighted strategies were used to calculate the influenced scores of genes based on the mutation constraints imposed by the network topology. The local weighted strategy considered the mutation frequency of the neighbors of seed nodes, whereas the global weighted strategy used an iterative propagation algorithm to evaluate the extent of genes influenced by mutation genes along the network. Ultimately, we calculated a mutation-based pathway enrichment score to reflect the accumulative effect of mutation genes on each pathway.

### Local weighted strategy

A mutation gene with a high degree in the PPI network may play critical functional roles [39], which may be reinforced by its neighbor mutations. Considering the number of mutation genes in the neighbors of each seed node, we proposed a local weighted strategy to distinguish the importance of seed nodes for each sample. Specifically, suppose that the background network has a total of *N* genes, of which *K* are seed nodes under investigation in the sample. For a given seed node (${G}_i$), there are 2 variables to characterize if any ${G}_i$ may be reinforced by the mutations of its neighbors: the number of mutation genes in the neighbors of ${G}_i$, designated as ${X}_i$, and the number of neighbors of ${G}_i$, designated as ${M}_i$. Thus, ${X}_i$ will follow a hypergeometric distribution, and the formula is as follows:


(1) \begin{eqnarray*}
p\left( {{X}_i = x} \right) = \frac{{\left( {\begin{array}{@{}*{1}{c}@{}} {{M}_i}\\ x \end{array}} \right)\left( {\begin{array}{@{}*{1}{c}@{}} {N - {M}_i}\\ {K - x} \end{array}} \right)}}{{\left( {\begin{array}{@{}*{1}{c}@{}} N\\ K \end{array}} \right)}},{\mathrm{\ }}i = 1,2,{\mathrm{\ }} \cdots K
\end{eqnarray*}

For a seed ${G}_i$ in the sample, if it plays a more important function in the disease progression, the number of mutation genes in its neighbors will be significantly larger than the expectation $E( {{X}_i} )$, which can be calculated as


(2) \begin{eqnarray*}
E\left( {{X}_i} \right) = \frac{{{M}_iK}}{N}
\end{eqnarray*}

Thus, we applied a rescaled form of ${X}_i - E( {{X}_i} )$ to quantify the important strength of ${G}_i$ in the sample, which was defined as local weight ${W}_i$:


(3) \begin{eqnarray*}
{w}_i = {X}_i - E\left( {{X}_i} \right)
\end{eqnarray*}

(4) \begin{eqnarray*}
{W}_i = lo{g}_\alpha \left( {{w}_iI\left( {{w}_i} \right) + \alpha } \right)
\end{eqnarray*}

where *I* is an indicator function; if ${w}_i$ is greater than 0, then its value is equal to 1 or otherwise equal to 0; and $\alpha $ is a scalar base to guarantee the weight has a minimum value of 1 (here, we set $\alpha $ as 2).

### Global propagation-based weighted strategy

Mutation genes can impact not only the activities of their neighboring genes but also other genes in the network due to network topology. We applied a global propagation algorithm, random walk with restart (RWR), to estimate the probable influence of nodes in the network by seed nodes (mutation genes). The RWR algorithm mimics an iterative random walker that, at each time step in the graph, begins from a group of source nodes (here corresponding to mutation genes) and moves to its immediate neighbors or returns to the source nodes. This algorithm, which captures global relationships within a network, has been effectively used to discriminate disease genes previously [40]. Given that different seed nodes have different important strengths, we improved the RWR algorithm by assigning the initial seed nodes with different weights. The improved RWR model is as follows:


(5) \begin{eqnarray*}
{p}_{t + 1} = \left( {1 - c} \right)A{p}_t + c{p}_0
\end{eqnarray*}

where ${p}_0 = ( {p_0^1,\ p_0^2, \cdots p_0^N} )$ is the initial probability vector, which was constructed by assigning to each seed node with its local weight value and remaining nodes with 0, and then it was normalized to a unit vector that sums to 1. *A* is the column-normalized adjacency matrix of the PPI network; the parameter *c* is a certain probability of continuing the random walk or restarting from the restart set, which has been reported to have only a slight effect on the results when it varied between 0.1 and 0.9 [40–42], and we set *c* = 0.7 in the study. ${p}_t = ( {p_t^1,\ p_t^2, \cdots p_t^N} )$ is a vector containing visiting probabilities of all nodes in the network at time point *t*. It will reach a steady state at a certain number of iterations, which is obtained when the difference between ${p}_{t + 1}$ and ${p}_t$ falls below 10e-10. The ${p}_{t + 1}$ reflects the extent to which seed nodes influence other nodes in the network and whose elements are defined as global weights of nodes.

### Calculate single-sample mutation-based pathway enrichment score

For each sample, we constructed a gene list $L = ({g}_1,\, {g}_2,\ldots{g}_N)$ by ranking the genes according to the normalized global weights and mapped the pathways to the ranked gene list, respectively. We then calculated a single-sample mutation-based pathway enrichment score (ssMutPES) for each pathway, which reflects how much a pathway is overrepresented at the top of the ranked gene list *L*. The weighted Kolmogorov–Smirnov statistic was used to calculate the ssMutPES. In particular, we calculated the fraction of genes not in the pathway (${F}_{\textit{NotP}}$) and the fraction of genes in the pathway (${F}_{InP}$) weighted by their global weights at a given position *i* in the list *L*, and the formulas are as follows:


(6) \begin{eqnarray*}
{F}_{InP}\left( i \right) = \mathop \sum \nolimits_{\begin{array}{@{}*{1}{c}@{}} {{g}_j \in P}\\ {j \le i} \end{array}} \frac{{{{\left| {{r}_j} \right|}}^p}}{{{N}_R}}
\end{eqnarray*}

(7) \begin{eqnarray*}
{F}_{\textit{NotP}}\left( i \right) = \mathop \sum \nolimits_{\begin{array}{@{}*{1}{c}@{}} {{g}_j \notin P}\\ {j \le i} \end{array}} \frac{1}{{{N}_{\textit{NotP}}}}
\end{eqnarray*}

where ${N}_R = \mathop \sum \nolimits_{{g}_j \in P} {| {{r}_j} |}^p$, ${r}_j$ is the global weight of gene *j*, ${N}_{\textit{NotP}}$ represents the number of genes in the list *L* not in the pathway, and *p* controls the extent of gene global weight; we set *P* = 1 as default value. The ssMutPES of pathway *P* is determined by going along the list *L* from position *i*:


(8) \begin{eqnarray*}
\textit{ssMutPES} = ma{x}_{i \in L}\left( {{F}_{InP}\left( i \right) - {F}_{\textit{NotP}}\left( i \right)} \right)
\end{eqnarray*}

A pathway with a large ssMutPES value indicates the pathway located at the very top of the list *L*, suggesting the pathway activity may tend to be induced by the mutation genes. Thus, we refer to the ssMutPES as mutation-induced pathway activity hereafter. To prevent any potential confusion, we assigned the ssMutPES of the pathway as zero if its ssMutPES <0, which indicates the mutation genes have a slight effect on the pathway.

### Identifying cancer subtypes

To assess the performance of the ssMutPA approach in real-world data analysis, we applied it to 33 different cancer types in TCGA to determine prognosis-related subtypes. For each cancer type, we first identified the prognostic pathways using the univariate Cox proportional hazards regression model and then constructed a patient–patient similarity matrix based on the Euclidean distance between pathway activities of samples. The spectral clustering algorithm, which has stronger adaptability to data distribution and an excellent clustering effect, was used to classify samples into different subtypes. We applied the “specc” function from the “kernlab” package [43] to implement this algorithm. To ascertain the optimal number of clusters, we employed the algorithm of the maximum value of the index, executed by the “Nbclust” function from the “Nbclust” package [44], and we set the “index” parameter as “silhouette,” which avoids overclustering and produces clusters with a very small sample size. Moreover, the Kaplan–Meier curve analysis and log-rank test were employed to test the prognostic differences among subtypes.

## Results

### Performance of ssMutPA in cancer stratification

**Figure 1:**
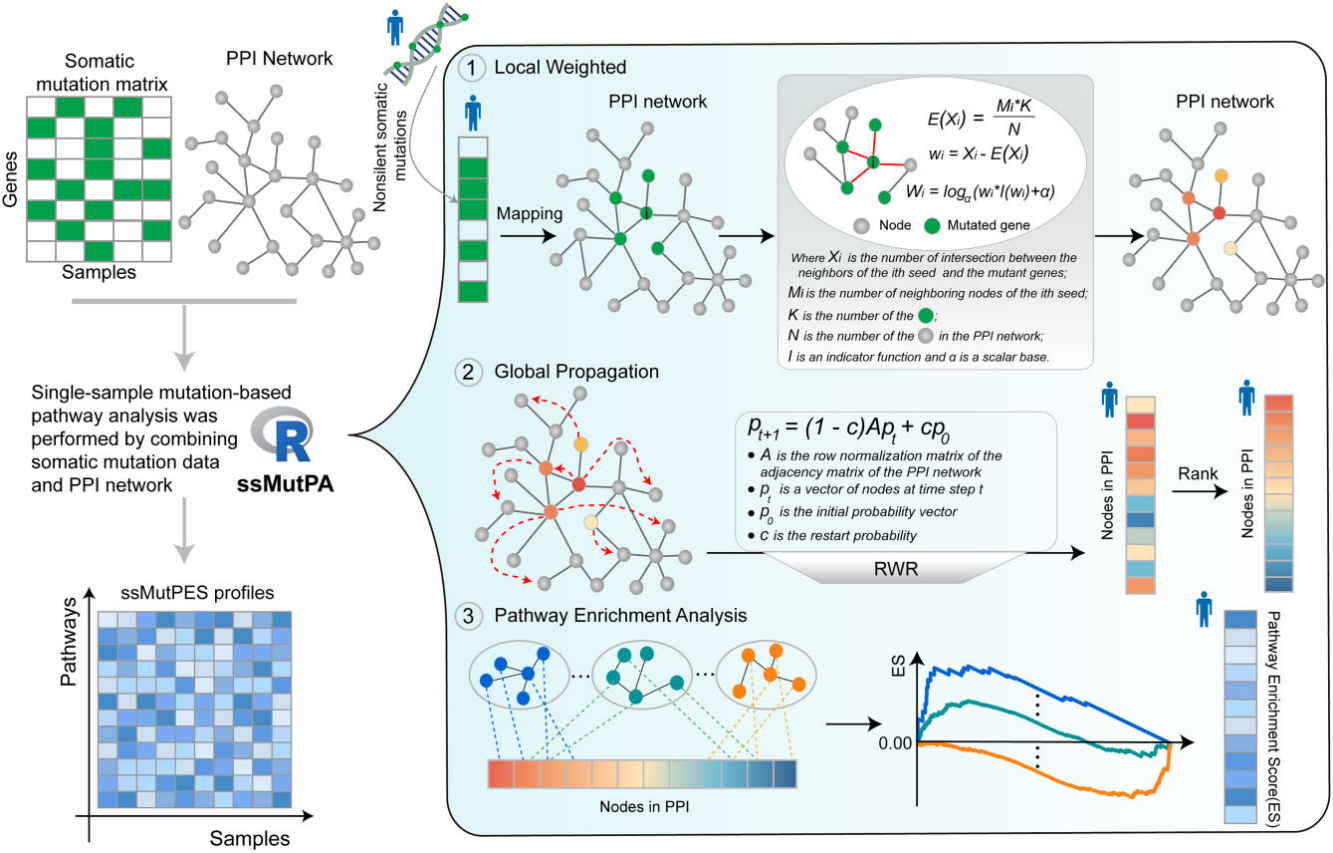
The workflow of the ssMutPA method.

Recently, single-sample pathway (or gene set) enrichment analysis approaches have received extensive attention and promoted the development of precision medicine. However, these approaches mainly focus on gene expression data and do not consider gene mutation information. In the study, we proposed a novel ssMutPA approach that uses network-based local and global weighted strategies to calculate the ssMutPES, reflecting mutation-based pathway activity. The detailed framework of ssMutPA is shown in Fig. 1.

**Figure 2:**
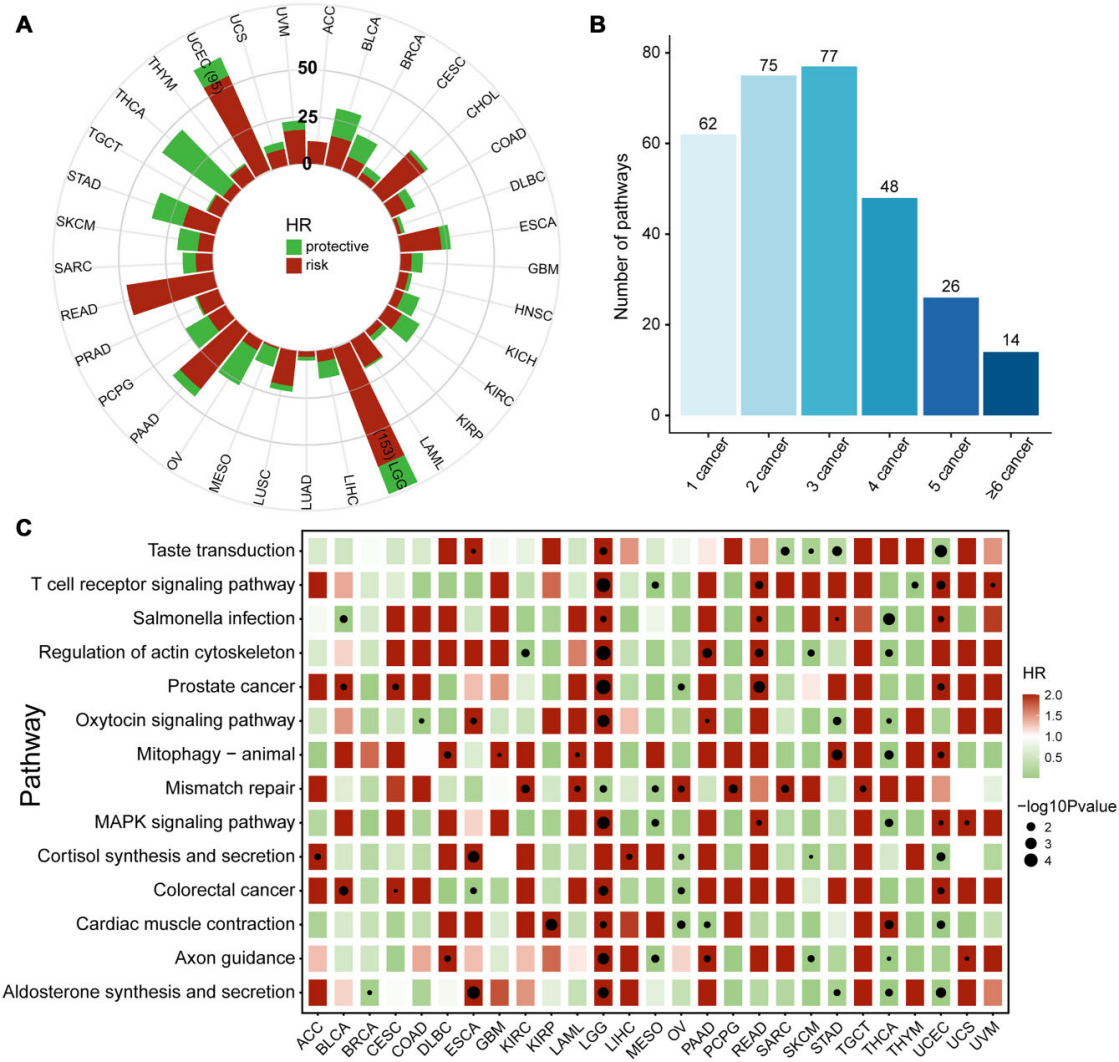
Overview of the prognostic-relevant pathways identified by ssMutPA in pan-cancer. (A) The number of risk/protective pathways identified in 33 cancer types. (B) The number of overlapped prognostic-relevant pathways. (C) Dot plot of univariate HRs and *P* values for the overlapped pathways in corresponding cancers. The color indicates the value of the HR, and the circle size represents the significance of *P* values.

To test whether ssMutPA could effectively identify aberrant pathways associated with clinical prognosis, we applied it to 33 cancer types from the TCGA database. For each cancer, we calculated ssMutPESs of 323 KEGG pathways and then performed survival analysis using the univariate Cox proportional hazards regression. It was shown that the protective (Hazard Ratio (HR) < 1) and risk (HR > 1) prognostic pathways (Cox *P* < 0.05) varied among the 33 different cancer types (Fig. 2A). Through comparing, we observed that most pathways are associated with only a minimal number of cancers; some of them are cancer specific (Fig. 2B), whereas there are 14 common pathways shared by at least 6 cancer types, including mismatch repair, T-cell receptor signaling pathway, regulation of actin cytoskeleton, MAPK signaling pathway, and so on (Fig. 2C). Most of these pathways have been reported by previous studies to be associated with the occurrence and progression of diseases. For example, mismatch repair is involved in DNA replication and gene recombination progress of cells in the human body and is essential for maintaining genome stability, and loss of mismatch repair function leads to microsatellite instability, which may affect disease prognosis or response to drugs [45, 46]. T cells play a pivotal role in the immune response and are part of the adaptive immune system that fights against a variety of infections and cancers [47, 48]. According to the ssMutPESs of these 2 pathways, we respectively performed Kaplan–Meier curve analysis and the log-rank test in their significant associated cancer types. For each pathway, we used the “surv_cutpoint” function in the “survminer” package [49] to determine the optimal cut-point of ssMutPESs. The results revealed that the patients in each corresponding cancer type could be classified into 2 subgroups with significant differences in overall survival (OS) (log-rank test, *P* < 0.05) (Supplementary Fig. S1A, B). Moreover, we found that the distributions of ssMutPESs between high-score and low-score subgroups presented significant differences (Supplementary Fig. S1C, D).

**Figure 3:**
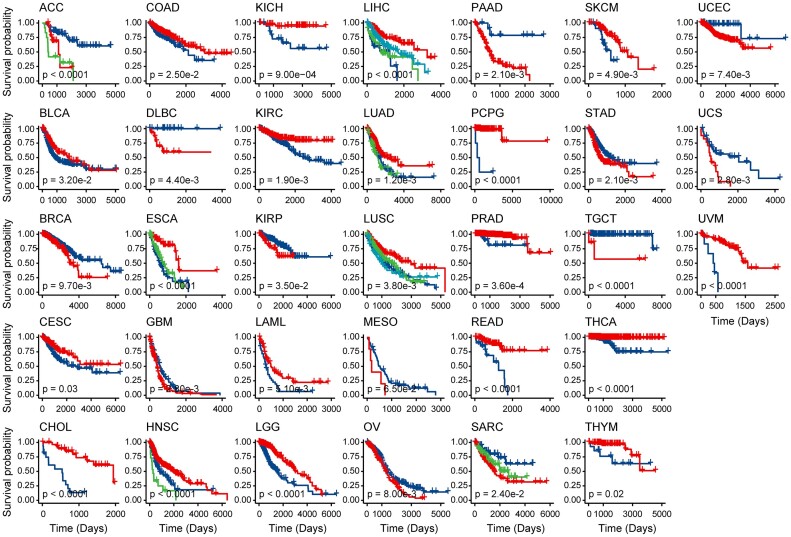
Kaplan–Meier survival curves of OS comparing the subtypes clustered based on the ssMutPES profiles across 33 cancer types from TCGA.

To test if the pathway ssMutPESs could stratify patients with cancer into clinically relevant subtypes, we performed an unsupervised spectral clustering algorithm on the ssMutPES profiles of prognostic pathways in each of the 33 cancer types. For each cancer type, we used the algorithm of the maximum value of the index to determine the relevant number of clusters (see Materials and Methods). We found that each cancer type could be stratified into 2 to 4 subtypes, and the subtypes exhibited significant differences in patient prognosis (OS, log-rank test, *P* < 0.05) across all cancer types except mesothelioma (MESO) (Fig. 3).

### Application of the ssMutPA approach in glioma

**Figure 4:**
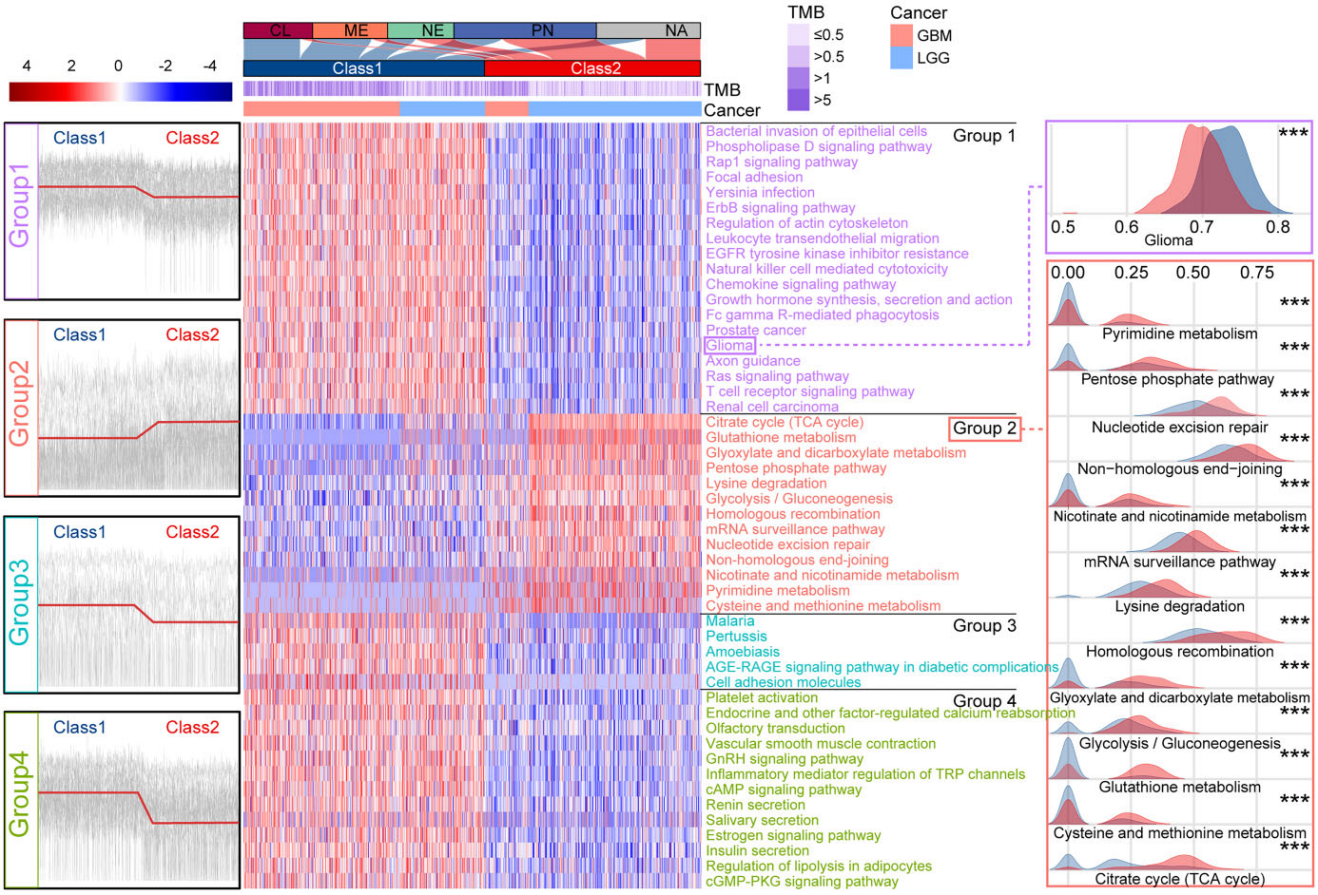
Heatmap of the ssMutPESs of the top 50 characteristic pathways in glioma. The line plots on the left of the heatmap illustrate the expression level of pathways in different groups within the 2 subtypes; the Sankey diagram above the heatmap displays the correspondence between clinically relevant subtypes and the subtypes we identified. On the right, we depicted ridge plots of ssMutPESs of pathways to reflect the distribution of these pathways in the 2 subtypes. Wilcoxon rank-sum test was used to assess the significance of the difference between subtypes: *P < 0.05, **P < 0.01, ***P < 0.001.

To illustrate the performance of ssMutPA in more detail, we applied it to glioma in TCGA. Glioma is one of the most common primary brain tumors and is usually associated with high morbidity and mortality. In TCGA, gliomas were categorized as glioblastoma multiforme (GBM) and brain lower grade glioma (LGG) in accordance with the degree of malignancy, whereas in this study, we merged the 2 datasets to systematically identify glioma subtypes and performed subsequent analyses. First, we performed univariate Cox proportional hazards regression analysis on the ssMutPES profiles for each pathway in GBM and LGG, and 215 pathways (Supplementary Table S4) associated with overall survival were identified (Cox *P* < 0.05). According to the ssMutPESs of these pathways, the patients were classified into 2 subtype clusters (classes 1 and 2) through the spectral clustering algorithm (Supplementary Fig. S2), and the top 50 most significant pathways were used to show our results in detail (Fig. 4). Through comparing the patients between the subtypes, it was found that the class 1 subtype primarily consists of patients with GBM, while the class 2 subtype mainly consists of patients with LGG. Moreover, we found that the pathways were clustered into 4 groups (groups 1 to 4). The pathways in group 2 primarily involved metabolic pathways such as glutathione metabolism, glyoxylate and dicarboxylate metabolism, pentose phosphate pathway, and so on, and the ssMutPESs of these pathways in the patients of the class 2 subtype are significantly higher than that of the class 1 subtype patients (Wilcoxon rank-sum test, *P* < 0.001). In contrast, the pathways in other groups (groups 1, 3, and 4) mainly included signaling pathways, and their ssMutPESs are significantly higher in the patients of the class 1 subtype compared with the class 2 subtype. More importantly, the glioma pathway was identified with higher ssMutPESs in the class 1 subtype patients, which indicates that the class 1 subtype patients accumulated with more gene mutations in the pathway (Fig. 4). These findings illustrated that class 1 subtype patients were characterized by mutation-induced signaling pathways, whereas the class 2 subtype patients were characterized by mutation-induced metabolic pathways.

**Figure 5:**
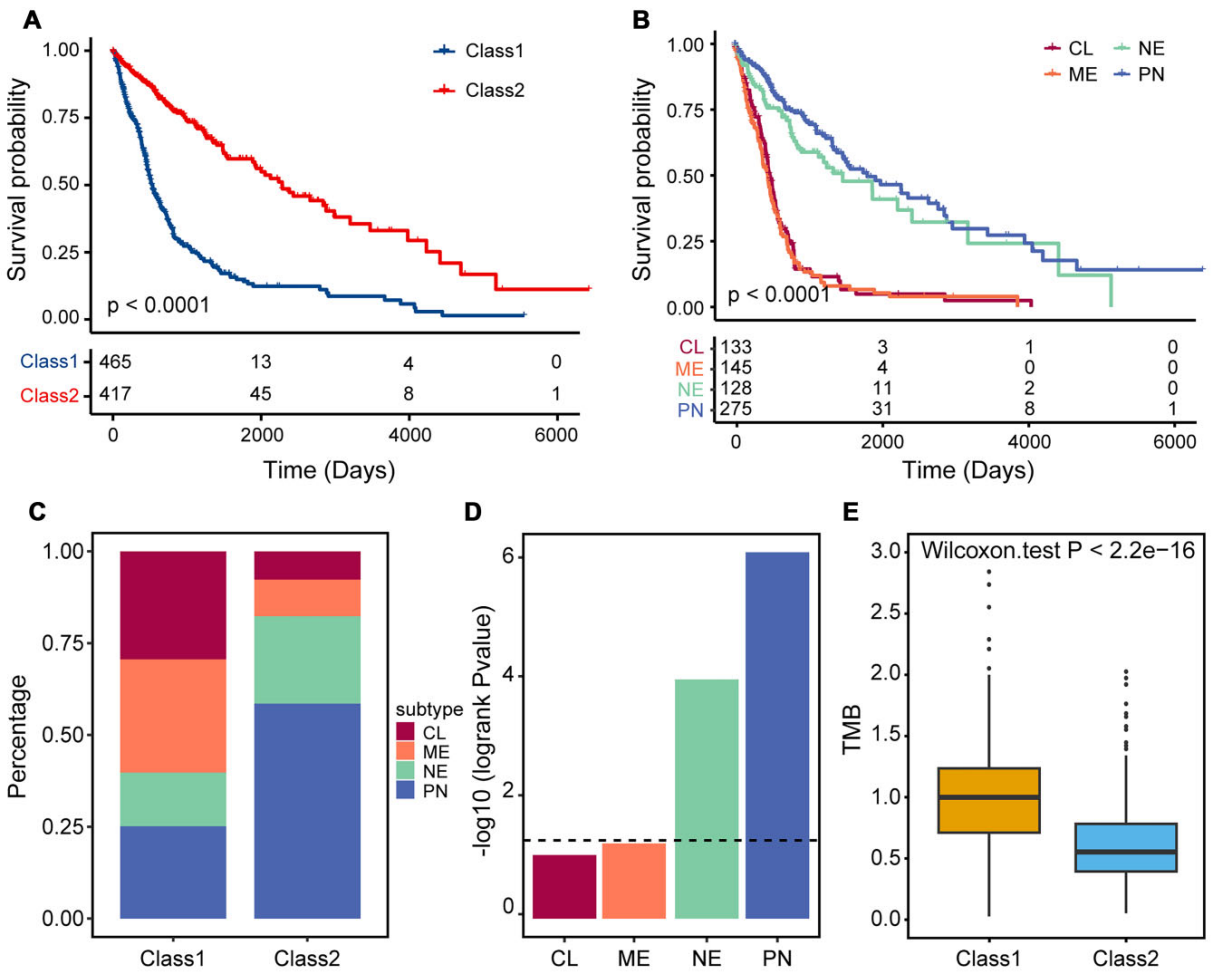
Comparison of the subtypes identified based on the ssMutPES profiles with clinically relevant subtypes in glioma. (A) Kaplan–Meier survival curves of OS comparing subtypes identified based on ssMutPES profiles. (B) Kaplan–Meier survival curves of OS comparing clinically relevant subtypes. (C) The proportion of the clinically relevant subtypes in class 1 and class 2 subtypes. (D) The *P* value of the log-rank test for clustering each clinically relevant subtype of patients using our method. The black dashed line represents *P* = 0.05. (E) Comparison of the TMB level between patients in 2 subtypes.

To assess the association of subtypes with clinical characteristics, we first performed survival analysis. The result showed that the patients of the class 2 subtype showed significantly better prognosis than the class 1 subtype (Kaplan–Meier survival analysis, log-rank test, *P* < 0.0001) (Fig. 5A). We then compared our subtypes with the clinically relevant glioma subtypes, including proneural (PN), neural (NE), classical (CL), and mesenchymal (ME) [50]. Survival analysis demonstrated that the OS of NE/PN subtypes was significantly longer than that of the CL/ME subtypes; however, samples across 4 different subtypes were not completely separated from each other (Fig. 5B). By comparing with our subtypes, we found that the CL/ME patients are mainly included in the class 1 subtype, while NE/PN patients are mainly included in the class 2 subtype (Figs. 4 and 5C). Moreover, in each original subtype cohort, we respectively performed survival analysis according to our subtypes. Interestingly, the patients in each original subtype could be classified into class 1 and class 2 groups (Fig. 5D and Supplementary Fig. S3). These results suggested that our pathway-based subtypes may complement the original subtypes and promote the development of precision medicine. Furthermore, we compared the tumor mutation burden (TMB) between class 1 and class 2 subtype patients and found that the class 1 subtype patients exhibited significantly larger TMB values than class 2 (Wilcoxon rank-sum test, *P* < 2.20e-16, Fig. 5E). This implied that the class 1 subtype patients were accumulated with more mutations, which resulted in poor prognosis.

Furthermore, we tested the differences in tumor microenvironment (TME)–related characteristics between class 1 and class 2 subtypes. Based on the gene expression data of the TCGA glioma patients, we calculated the TME cell infiltration levels according to the cell-type identification by estimating relative subsets of the RNA transcript (CIBERSORT) method [51] and found that macrophages (M0, M1, M2), CD8^+^ T cells, T follicular helper cells, and so on showed a significantly higher infiltration level in the class 1 subtype patients than that of the class 2 subtype patients (Supplementary Fig. S4A). We also evaluated the immune score, stromal score, and tumor purity with the ESTIMATE method [52]. Intriguingly, the immune score and stromal score were notably higher in the class 1 subtype compared with class 2 subtype (Wilcoxon rank-sum test, *P* < 0.001), while tumor purity exhibited the opposite result (Supplementary Fig. S4B–D). The above results indicated that the class 1 subtype patients generally presented higher immune activities.

Finally, to assess the generalizability and robustness of the ssMutPA method, we collected 2 independent glioma datasets (Varn et al. cohort and Wang et al. cohort) from cBioPortal. ssMutPA was respectively applied to these 2 datasets to calculate ssMutPES profiles, followed by the same process to identify significant prognosis-related pathways. Through comparing the pathways with the results from the TCGA glioma cohort, we observed 90.48% and 80.65% of the significant pathways identified in the Varn et al. cohort and Wang et al. cohort overlapped with those of in TCGA glioma cohort (Supplementary Fig. S4E). Moreover, we also performed the robustness analysis to test the influence of the network structure. Specifically, we randomly removed 5%, 10%, 15%, and 20% of the edges from the original network and recalculated the ssMutPES profiles for each removal and then identified the prognosis-related pathways based on the ssMutPES profiles. We found that the percentage of overlapped prognosis-related pathways to original significant pathways remained above 80%, even after the removal of up to 20% of the edges (Supplementary Fig. S4F).

### Identifying pathway-based cancer subtypes associated with response to ICIs

**Figure 6:**
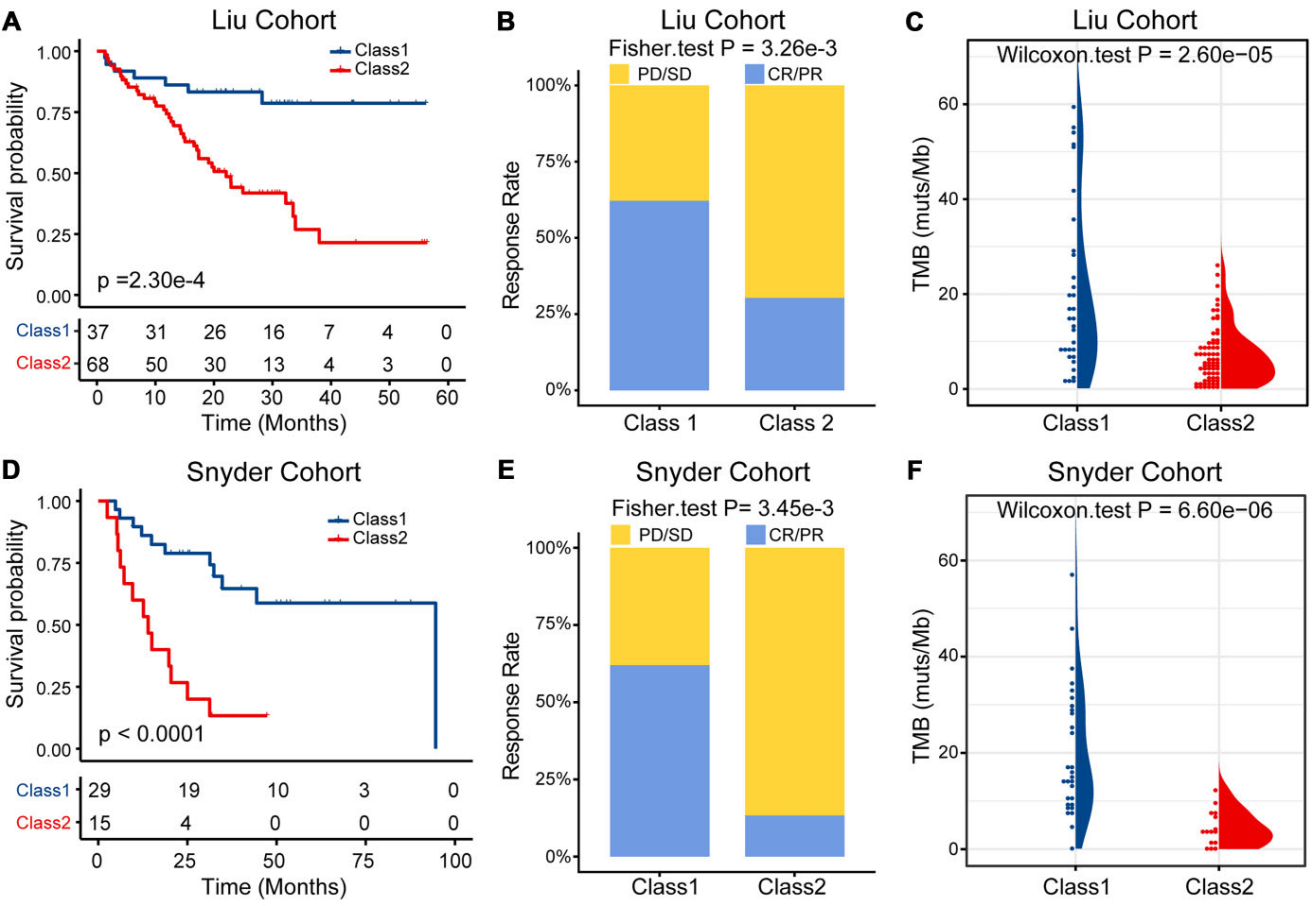
Identify pathway-based cancer subtypes in the immunotherapy datasets. (A) Kaplan–Meier survival curves of OS comparing the class 1 and class 2 groups from the Liu et al. cohort. (B) Comparison of the ORR between the class 1 and class 2 groups from the Liu et al. cohort. (C) Comparison of the TMB level between the class 1 and class 2 groups from the Liu et al. cohort. (D) Kaplan–Meier survival curves of OS comparing the class 1 and class 2 groups from the Snyder et al. cohort. (E) Comparison of the ORR between the class 1 and class 2 groups from the Snyder et al. cohort. (F) Comparison of the TMB level between the class 1 and class 2 groups from the Snyder et al. cohort.

To further test whether the ssMutPA approach could identify key pathways and cancer subtypes associated with response to ICIs, we applied ssMutPA to the Liu et al. cohort, comprising 105 patients with melanoma treated with the PD-1 inhibitor [33]. According to the ssMutPESs of pathways, 37 survival-related key pathways were identified with the univariate Cox proportional hazards regression analysis (*P* < 0.05). Based on the ssMutPESs of these pathways, 2 subtypes (class 1 and class 2) were obtained by using the spectral clustering algorithm. We found that the class 1 subtype patients presented a longer OS (log-rank test, *P* = 2.30e-04, Fig. 6A) and a higher objective response rate (ORR) than the class 2 subtype patients (Fisher exact test, *P* = 3.26e-03, Fig. 6B). Among the identified key pathways, several were immune-related, such as the T-cell receptor signaling pathway and cellular senescence. We then detected the mutation patterns of the top 20 genes in terms of mutation rate within the T-cell receptor signaling pathway. The results showed that the mutation rates of these genes in the class 1 subtype patients were obviously higher than the class 2 subtype (Supplementary Fig. S5A). Additionally, we compared TMB between class 1 and class 2 subtypes and found that the patients of the class 1 subtype presented higher TMB values than the class 2 subtype (Wilcoxon rank-sum test, *P* < 2.60e-05, Fig. 6C). Moreover, we applied the ssMutPA approach to the Snyder et al. cohort, comprising 44 patients with melanoma treated with CTLA-4 [34]. Following the same analysis as described above, 9 key pathways associated with OS were found, and the patients with melanoma were also clustered into 2 distinct subtypes. Consistently, the patients of the class 1 subtype presented a longer overall survival (log-rank test, *P* < 0.0001, Fig. 6D) and a higher ORR than the patients of the class 2 subtype (Fisher exact test, *P* = 3.45e-03, Fig. 6E). Investigating the key pathways, some important pathways, such as the IL-17 signaling pathway and ECM–receptor interaction, were identified and have been reported to be frequently activated or mutated in cancer. By comparing the mutation status of the top 20 genes in the IL-17 signaling pathway between class 1 and class 2 subtypes, we found that these genes are more frequently mutated in the class 1 subtype (Supplementary Fig. S5B). Finally, we also found that the class 1 subtype patients showed a higher TMB than the class 2 subtype (Wilcoxon rank-sum test, *P* < 6.60e-06, Fig. 6F). These results illustrate that the ssMutPA approach could effectively identify mutation-induced aberrant pathways and cluster patients with melanoma into subtypes with different prognoses and immunotherapy responses.

### Comparison of ssMutPA with other individualized pathway activity analysis methods based on transcriptomic data

**Figure 7:**
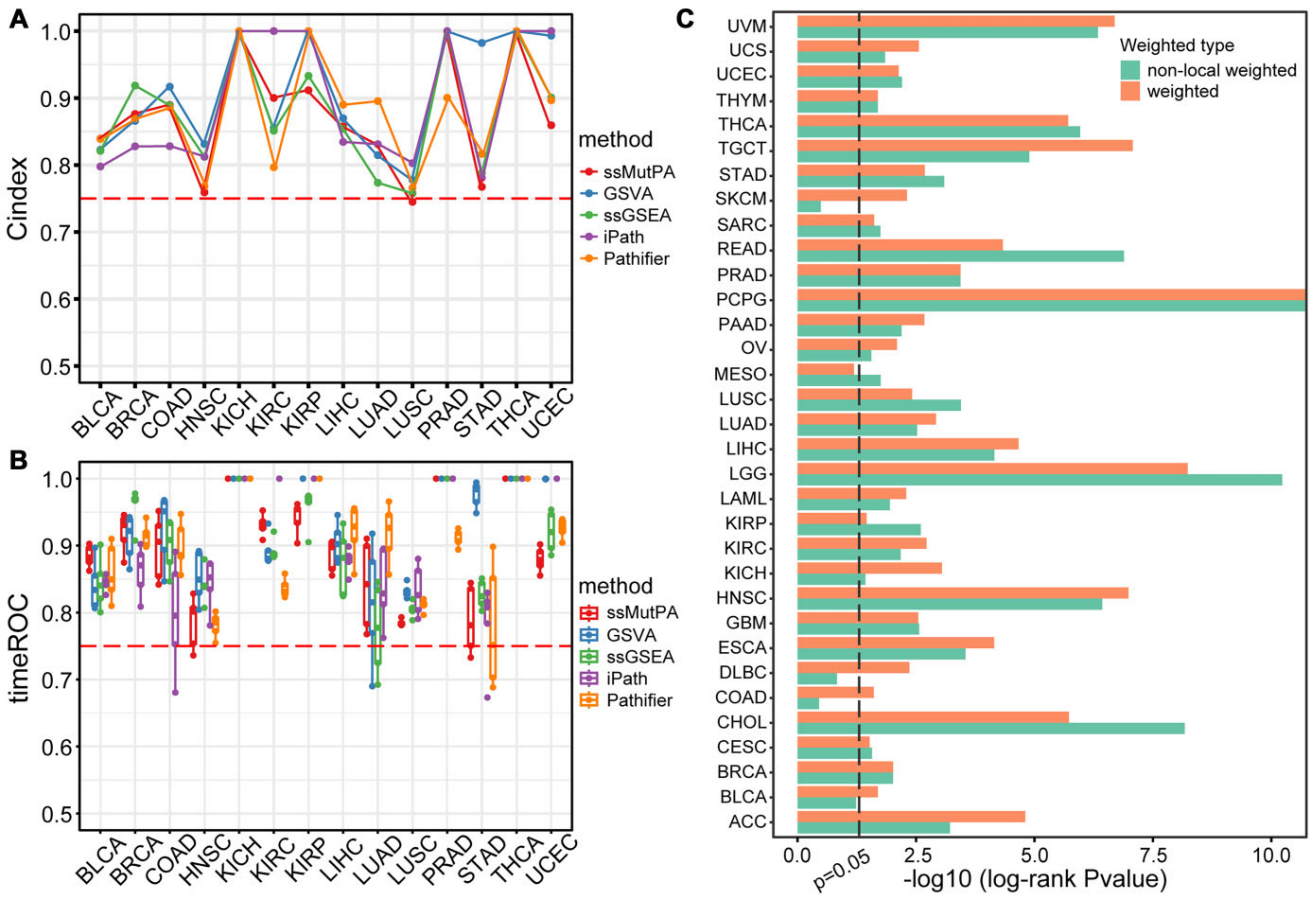
Comparison of ssMutPA with other methods. (A, B) Performance comparison of ssMutPA with other pathway activity algorithms on survival prediction. (A) The C-index of different methods across 14 cancer types. (B) Time-dependent AUC of 1–5 years for different methods across 14 cancer types. (C) Comparing the clustering performance (*P* value of the log-rank test) of ssMutPA with nonlocal weighted ssMutPA across 33 cancer types.

To explain whether the ssMutPA approach could provide new biological insights, we compared it with other individualized pathway activity analysis methods, including GSVA, ssGSEA, iPath, and Pathifier. As some of these methods require normal samples to infer pathway activities, we used 14 cancer types from TCGA (BLCA, BRCA, COAD, etc.), each of which includes at least 20 normal samples. For a fair comparison, we examined the prognostic prediction performance of each method. Specifically, we respectively applied these methods to each cancer dataset to obtain individualized pathway activity profiles. We then used the forward-stepwise algorithm to determine the optimal prognostic pathway sets with the highest predictive power (the concordance index, C-index, was used) and constructed a pathway-based prognostic signature according to the multivariate Cox proportional hazards regression model. Comparing the pathway-based prognostic signatures of each method, we found that the C-index of the ssMutPA approach was greater than or equal to that of the other methods across most of the 14 cancer types (Fig. 7A). Additionally, we performed time-dependent receiver operating characteristic (ROC) curve analysis for 1–5 years for each method’s signature. The results showed that the values of area under the ROC curve (AUROC) of the ssMutPA signature exceeded 0.75 in almost all cancers, which were also comparable to the signatures of other methods (Fig. 7B and Supplementary Fig. S6A). Finally, we applied ssGSEA, GSVA, iPath, and Pathifier to TCGA glioma (LGG and GBM) gene expression data to calculate single-sample pathway activity profiles and identify prognosis-related pathways through univariate Cox proportional hazards regression analysis. Comparing these results with the top 20 pathways identified by ssMutPA, we found that ssMutPA uniquely identified 14 pathways associated with patient prognosis (Supplementary Table S5). Most of these pathways, such as the citrate cycle (TCA cycle) [53] and Rap1 signaling pathway [54], have been confirmed to be associated with glioma prognosis in previous studies. These results illustrated that the signature of ssMutPA could effectively predict the prognosis of patients with cancer. More importantly, the ssMutPA method uses gene mutation data to calculate the pathway activities (ssMutPES), distinct from other methods that use gene expression data. Therefore, the ssMutPA method may provide some new insight into inferring individualized pathway activity and complement the current methods focused on gene expression data.

### Comparison of ssMutPA with nonweighted ssMutPA

Considering that the effect of mutation genes may be reinforced by their neighbors in the PPI network, we proposed a novel local weighted strategy to distinguish the importance of mutation genes for each sample. To assess if the local weighted strategy increases the performance of ssMutPA, we compared the clustering results of ssMutPA with that of ssMutPA without local weight (expressed as nonlocal weighted ssMutPA) across 33 cancers in TCGA. Specifically, we applied nonlocal weighted ssMutPA to each cancer type and obtained the ssMutPES profiles of pathways. Subsequently, the same procedure was performed on the ssMutPES profiles to identify cancer subtypes. By comparing the prognostic difference among subtypes (Kaplan–Meier survival analysis), we found that the performance (*P* value of the log-rank test) of ssMutPA outperformed nonlocal weighted ssMutPA in almost all cancer types (Fig. 7C). Moreover, in addition to local weighted, we introduced a global propagation-based weighted strategy to assess the impact of mutated genes on the activity of other genes within the network. To evaluate whether the global propagation strategy enhanced the performance of ssMutPA, we compared the clustering results of ssMutPA with the ssMutPA without global weighted (expressed as nonglobal weighted ssMutPA) across 33 cancer types from the TCGA database. After excluding the global propagation-based weights, we recalculated ssMutPES using the hypergeometric distribution test. Following the same comparison process, we found that ssMutPA’s performance (log-rank test *P* values) still outperformed nonglobal weighted ssMutPA (Supplementary Fig. S6B). These results demonstrated that the local and global weighted strategies were essential to ssMutPA, which increases its predicted efficacy.

## Discussion

Due to the high heterogeneity of cancer, the gene-level biomarkers were generally limited by the instability. The pathways reflecting the key biological processes and cellular functions could help to identify more effective and reproducible biomarkers. Therefore, an increasing number of single-sample pathway activity calculation methods and tools are being developed for identifying dysregulated pathways in complex diseases [14–17]. However, almost all methods focus on gene expression data, overlooking the gene mutation data because of their sparseness and discreteness. In this study, ssMutPA was developed to infer individualized pathway activities by integrating somatic mutation data and PPI network topology. To demonstrate the effectiveness of ssMutPA, we applied it to 33 cancer types from the TCGA database. Based on the mutation-induced pathway activity (ssMutPES) profiles, the patients could be clustered into different subtypes with significantly different prognoses in each cancer type. When comparing the ssMutPA approach with other individualized pathway activity analysis methods, including GSVA, ssGSEA, iPath, and Pathifier, we found that the prognosis prediction power of the ssMutPA-based signature was superior to other methods. This indicated that the mutation-based individualized pathway analysis may complement the existing methods focused on gene expression data and provide some new insights into cancer precision medicine.

Because of the sparseness and discreteness of mutations, we mapped them to the PPI network to evaluate the effect of mutations on network genes. As the different mutation genes generally possess different network topology, they may perform different influences on diseases. We thus proposed a novel local weighted strategy, which takes into account the difference in network topology and the number of mutated genes in the neighbor of each seed node, to determine the importance of mutation genes in every sample (see Materials and Methods). This strategy not only emphasizes the importance of mutated genes themselves but also indicates the degree to which the mutated genes are affected by neighbor nodes in the network. It is particularly meaningful for our ssMutPA method. To demonstrate the importance of the local weight, we compared the ssMutPA method with the method without local weight (defined as nonweighted ssMutPA), and the results showed that the ssMutPA method was superior to the nonweighted ssMutPA method in the clustering performance (Fig. 7C). This indicated that the local weighted strategy can improve the performance of the method and is crucial for the ssMutPA method. Although we demonstrated the robustness of the ssMutPA method to the network structure, the method may also limited by the incomplete PPI network. With the PPI network constantly updated, the applicability of the ssMutPA method will be further enhanced.

In summary, this study presents a novel ssMutPA method for inferring individualized pathway activities by integrating somatic mutation data and the PPI network. The mutation-based individualized pathway activity profiles could effectively reveal patient stratification with significantly different prognoses. Moreover, the ssMutPA outperformed the current individualized pathway analysis methods focused on gene expression data in prognostic prediction performance and thus may complement these methods. Finally, we implemented ssMutPA as an R-based software package, which is available at [28].

## Availability of Source Code and Requirements

Project name: ssMutPA.

Project homepage: https://CRAN.R-project.org/package=ssMutPA

Operating system(s): Platform independent

Programming language: R 4.0.0 or higher

Other requirements: R packages ggplot2, ggridges, grDevices, igraph, kernlab, maftools, Matrix, NbClust, parallel, pheatmap, RColorBrewer, stats, survival, utils

License: GPL 2.0 or higher

BioTools ID: biotools: ssMutPA

RRID: SCR_025644.

## Supplementary Material

giae105_GIGA-D-24-00212_Original_Submission

giae105_GIGA-D-24-00212_Revision_1

giae105_Response_to_Reviewer_Comments_Original_Submission

giae105_Reviewer_1_Report_Original_Submission Xionghui Zhou -- 6/26/2024 Reviewed

giae105_Reviewer_1_Report_Revision_1 Xionghui Zhou -- 10/22/2024 Reviewed

giae105_Reviewer_2_Report_Original_Submission Sihan Chen -- 8/5/2024 Reviewed

giae105_Reviewer_3_Report_Original_Submission Je-Keun Rhee -- 8/11/2024 Reviewed

giae105_Reviewer_3_Report_Revision_1 Je-Keun Rhee -- 11/1/2024 Reviewed

giae105_Supplemental_Files

## Data Availability

The details of the patient cohorts used for pan-cancer analysis and case studies in this study are listed in Supplementary Tables S1–S3. The pathways used for ssMutPA are obtained from the KEGG database. The integrated PPI network and the core code implemented for ssMutPA are included in the R package ssMutPA, which is freely available on CRAN [28]. All supporting data and materials are available in the *GigaScience* database, GigaDB [55].

## Additional Files

**Supplementary Fig. S1**. Individual pathway prognostic analysis.

**Supplementary Fig. S2**. Heatmap of the characteristic pathways in glioma. The Sankey diagram above the heatmap displays the correspondence between transcriptome subtypes and the subtypes we identified.

**Supplementary Fig. S3**. Further stratification of clinically relevant subtype patients based on ssMutPA-determined subtypes. Kaplan–Meier survival curves of OS comparing the class 1 and class 2 patients within clinically relevant subtypes (CL, NE, PN, ME).

**Supplementary Fig. S4**. Analysis of immune features between glioma subtypes and robustness assessment of the ssMutPA method.

**Supplementary Fig. S5**. Mutation analysis of genes involved in the characteristic pathways.

**Supplementary Fig. S6**. Comparison of ssMutPA with other methods.

**Supplementary Table S1**. The information of all the cohorts we used in this study.

**Supplementary Table S2**. Detailed information on 33 cancer types in the TCGA database.

**Supplementary Table S3**. The accession identifiers for all samples.

**Supplementary Table S4**. Prognostically relevant pathway identified in glioma (Cox *P* < 0.05).

**Supplementary Table S5**. Comparison of top 20 pathways identified by ssMutPA, ssGSEA, GSVA, iPath, and Pathifier in glioma.

## Abbreviations

AUROC: area under the receiver operating characteristic curve; CIBERSORT: cell-type identification by estimating relative subsets of RNA transcripts; C-index: concordance index; CL: classical; CR: complete response; CTLA-4: T-lymphocyte-associate antigen 4; GBM: glioblastoma multiforme; GSEA: gene set enrichment analysis; GSVA: gene set variation analysis; ICIs: immune checkpoint inhibitors; KEGG: Kyoto Encyclopedia of Genes and Genomes; LB: long benefit; LGG: lower-grade glioma; MAF: Mutation Annotation Format; ME: mesenchymal; MESO: mesothelioma; NB: nonbenefit; NE: neural; ORR: objective response rate; OS: overall survival; PD: progressive disease; PD-1: programmed cell death protein 1; PEA: pathway enrichment analysis; PN: proneural; PPI: protein–protein interaction; PR: partial response; ROC: receiver operating characteristic; RWR: random walk with restart; SD: stable disease; SPIA: signaling pathway impact analysis; ssGSEA: single-sample gene set enrichment analysis; ssMutPA: single-sample mutation-based pathway analysis; ssMutPES: single-sample mutation-based pathway enrichment score; TCGA: The Cancer Genome Atlas; TMB: tumor mutation burden; TME: tumor microenvironment; HR: Hazard Ratio.
