# Supplementary material for: ssMutPA: single-sample mutation-based pathway analysis approach for cancer precision medicine
Source: Gigascience. 2024 Dec 20;13:giae105. doi: 10.1093/gigascience/giae105 (PMC11659979; doi:10.1093/gigascience/giae105)
Supplement: giae105_GIGA-D-24-00212_Original_Submission [file giae105_giga-d-24-00212_original_submission.pdf]

# GigaScience


## ssMutPA: Single-sample Mutation-based Pathway Analysis approach for cancer precision medicine

--Manuscript Draft--

| | |
|---|---|
| **Manuscript Number:** | GIGA-D-24-00212 |
| **Full Title:** | ssMutPA: Single-sample Mutation-based Pathway Analysis approach for cancer precision medicine |
| **Article Type:** | Technical Note |
| **Funding Information:** | National Natural Science Foundation of China (62072145) — Dr. Junwei Han<br>National Natural Science Foundation of China (62372143) — Dr. Junwei Han<br>Natural Science Foundation of Heilongjiang Province (LH2019C042) — Dr. Junwei Han |
| **Abstract:** | Background<br><br>Single-sample pathway enrichment analysis is an effective approach for identifying cancer subtypes and pathway biomarkers, facilitating the development of precision medicine. However, the existing approaches focused on investigating the changes in gene expression levels but neglected somatic mutations which play a crucial role in cancer development.<br><br>Findings<br><br>In this study, we proposed a novel single-sample mutation-based pathway analysis approach (ssMutPA) to infer individualized pathway activities by integrating somatic mutation data and the protein-protein interaction (PPI) network. For each sample, ssMutPA first uses local and global weighted strategies to evaluate the effects of genes from mutations according to the network topology and then calculates a single-sample mutation-based pathway enrichment score (ssMutPES) to reflect the accumulated effect of mutations of each pathway. To illustrate the performance of ssMutPA, we applied it to 33 cancer cohorts from the TCGA database and revealed patient stratification with significantly different prognosis in each cancer type based on the ssMutPES profiles. We also found that the identified characteristic pathways with high overlap across different cancers could be used as potential prognosis biomarkers. Moreover, we applied ssMutPA to two melanoma cohorts with immunotherapy and identified a subgroup of patients who may benefit from therapy.<br><br>Conclusions<br><br>We provided evidence that ssMutPA could infer mutation-based individualized pathway activity profiles and complement the current individualized pathway analysis approaches focused on gene expression data, which may offer the potential for the development of precision medicine. ssMutPA is available at (https://CRAN.R-project.org/package=ssMutPA). |
| **Corresponding Author:** | Junwei Han<br>Harbin Medical University<br>Harbin, China CHINA |
| **Corresponding Author Secondary Information:** | |
| **Corresponding Author's Institution:** | Harbin Medical University |
| **Corresponding Author's Secondary Institution:** | |
| **First Author:** | Yalan He |


*Powered by Editorial Manager® and ProduXion Manager® from Aries Systems Corporation*

| | |
|---|---|
| **First Author Secondary Information:** | |
| **Order of Authors:** | Yalan He |
| | Qian Wang |
| | Jiyin Lai |
| | Bingyue Pan |
| | Siyuan Li |
| | Xilong Zhao |
| | Junwei Han |
| **Order of Authors Secondary Information:** | |
| **Additional Information:** | |
| **Question** | **Response** |
| Are you submitting this manuscript to a special series or article collection? | No |
| **Experimental design and statistics**<br><br>Full details of the experimental design and statistical methods used should be given in the Methods section, as detailed in our Minimum Standards Reporting Checklist. Information essential to interpreting the data presented should be made available in the figure legends.<br><br>Have you included all the information requested in your manuscript? | Yes |
| **Resources**<br><br>A description of all resources used, including antibodies, cell lines, animals and software tools, with enough information to allow them to be uniquely identified, should be included in the Methods section. Authors are strongly encouraged to cite Research Resource Identifiers (RRIDs) for antibodies, model organisms and tools, where possible.<br><br>Have you included the information requested as detailed in our Minimum Standards Reporting Checklist? | Yes |
| **Availability of data and materials** | Yes |

Powered by Editorial Manager® and ProduXion Manager® from Aries Systems Corporation

| | |
|---|---|
| All datasets and code on which the conclusions of the paper rely must be either included in your submission or deposited in publicly available repositories (where available and ethically appropriate), referencing such data using a unique identifier in the references and in the "Availability of Data and Materials" section of your manuscript.<br><br>Have you have met the above requirement as detailed in our Minimum Standards Reporting Checklist? | |

Manuscript Click here to access/download;Manuscript;manuscript.docx

# ssMutPA: Single-sample Mutation-based Pathway Analysis approach for cancer precision medicine

Yalan He[1,†], Qian Wang[1,†], Jiyin Lai [1,†], Bingyue Pan[1], Siyuan Li[1], Xilong Zhao[1], Junwei Han[1,*]

[1] College of Bioinformatics Science and Technology, Harbin Medical University, Harbin 150081, China.

† The authors should be regarded as joint First Authors.

* Corresponding Author: Junwei Han, College of Bioinformatics Science and Technology, Harbin Medical University, Harbin 150081, China, E-mail: hanjunwei@ems.hrbmu.edu.cn

## Abstract

**Background:** Single-sample pathway enrichment analysis is an effective approach for identifying cancer subtypes and pathway biomarkers, facilitating the development of precision medicine. However, the existing approaches focused on investigating the changes in gene expression levels but neglected somatic mutations which play a crucial role in cancer development.

**Findings:** In this study, we proposed a novel single-sample mutation-based pathway analysis approach (ssMutPA) to infer individualized pathway activities by integrating somatic mutation data and the protein-protein interaction (PPI) network. For each sample, ssMutPA first uses local and global weighted strategies to evaluate the effects of genes from mutations according to the network topology and then calculates a single-sample mutation-based pathway enrichment score (ssMutPES) to reflect the accumulated effect of mutations of each pathway. To illustrate the performance of ssMutPA, we applied it to 33 cancer cohorts from the TCGA database and revealed patient stratification with significantly different prognosis in each cancer type based on the ssMutPES profiles. We also found that the identified characteristic pathways with high overlap across different cancers could be used as potential prognosis biomarkers. Moreover, we applied ssMutPA to two melanoma cohorts with immunotherapy and identified a subgroup of patients who may benefit from therapy.

**Conclusions:** We provided evidence that ssMutPA could infer mutation-based individualized pathway activity profiles and complement the current individualized pathway analysis approaches focused on gene expression data, which may offer the potential for the development of precision medicine. ssMutPA is available at (https://CRAN.R-project.org/package=ssMutPA).

**Keywords:** somatic mutation; network topology; single-sample pathway analysis; cancer subtypes; precision medicine

## Background

Over the past decades, gene signatures derived from transcriptomics data have been recognized in multiple cancers [1-5]. However, their clinical application has been hindered by low reproducibility and small overlap [6-8]. Recently, many studies have proved that cancers are essentially caused by disturbances in the complex regulatory relationships among multiple functional genes, suggesting the need to convert gene expression data into pathway-level activity values for further studies [9, 10].

Pathway enrichment analysis (PEA) is currently the most popular method to interpret transcriptomics data using knowledge of gene sets or biological pathways. Thus, more and more PEA algorithms have been proposed for identifying biomarkers and cancer subtypes, such as Gene Set Enrichment Analysis (GSEA) [11], Signaling Pathway Impact Analysis (SPIA) [12], and CTpathway [13]. While these methods converted traditional gene expression data into pathway-level analysis and explored the dysregulated pathways between the two phenotypes, their effectiveness relied on a large number of sample data. More importantly, they ignore individualized patient information on pathways. To overcome these limitations, individualized pathway activity calculation methods and tools have been evolved, such as single-sample GSEA (ssGSEA) [14], Gene Set Variation Analysis (GSVA) [15], iPath [16], Pathifier [17], etc. ssGSEA was introduced by Barbie et al., who extended the GSEA algorithm to the single-sample level and calculated the enrichment statistic for each pathway on a single sample, thus reflecting the extent to which the genes contained in a particular pathway are up- or down-regulated in each sample. Similarly, GSVA estimated changes in pathway activity on a sample via an unsupervised manner, which first calculated the expression statistics of the

kernel estimates on a sample and subsequently figured up the activity of each pathway in a single sample, providing greater power to detect subtle changes in pathway activity across sample populations. Su et al. developed the iPath algorithm to classify tumor samples into two distinct groups by calculating pathway-based individual-level enrichment scores. The algorithm was applied in the pan-cancer analysis, and the results showed that iPath could effectively identify the pathway markers associated with overall survival, subtype, and stage of cancers. In Pathifier, for a tumor sample, a dysregulation score was assigned to each pathway by estimating the extent to which the pathway in that sample deviated from the normal samples, thereby reflecting the pathway's activity. All the methods described above rely on gene expression data, which is quite dynamic due to batch effects and many other factors [18, 19]. These problems would contribute to some extent to the low reliability and poor clinical applicability of the markers identified by these methods. Therefore, there is an urgent need to develop more comprehensive methods for individualized pathway analysis using other omics data.

Modern medicine has proved that the accumulation of genetic mutations is an important cause of cancers and plays an essential role in the occurrence and development of cancers [20-22]. In clinical practice, the mutation data was used far more commonly than other omics analyses. Although many mutation-based biomarkers have been identified in recent studies [23-26], they are difficult to consider the combined effects of the mutated genes in pathways for a single sample because of the sparseness and discreteness of mutation data. Therefore, the development of mutation-based individualized pathway analysis is urgently needed to identify cancer subtypes and biomarkers for precise treatment of patients.

Here, we innovatively proposed an approach called single-sample mutation-based pathway analysis (ssMutPA), which integrated somatic mutation data and the PPI network topology to infer individualized pathway activity profiles induced by mutations. ssMutPA considered the

positions of mutation genes in the PPI network for each sample and used local and global weighted strategies to evaluate the potential influence extent of genes from mutations. Then, it calculated the mutation-based pathway enrichment scores to reflect the individualized pathway activities induced by mutations. This will complement the current individualized pathway analysis approaches focus on gene expression data and provide something new insight into the initiation and progression of cancer. We applied ssMutPA to 33 cancers from the TCGA database and identified cancer subtypes with significant prognostic differences in 32 cancer types. The identified coherent pathways across these cancers could be used as effective prognostic biomarkers. In addition, by applying ssMutPA to two melanoma cohorts treated with immune checkpoint inhibitors (ICIs), the patients were classified into two subgroups with significantly different immunotherapy responses based on pathway activity profiles. Ultimately, to facilitate the use of our method, ssMutPA was developed as an R-based package, which is freely available on the Comprehensive R Archive Network (CRAN) (https://CRAN.R-project.org/package=ssMutPA).

## Materials and Methods

### Data collection and processing

To demonstrate the effectiveness and applicability of ssMutPA, we downloaded somatic mutation data and corresponding clinical information for 33 cancer types from The Cancer Genome Atlas (TCGA) database (https://portal.gdc.cancer.gov/) [27]. In this study, we focused exclusively on non-silent mutations extracted from Mutation Annotation Format (MAF) files. For each cancer type, we used primary tumor samples for our analysis. To further explore whether our approach can be applied to patients treated with ICIs, we collected two datasets treated with ICIs from the cBioPortal database (https://www.cbioportal.org/) and published literature [28, 29]. The Liu et al. dataset contains somatic mutation data and clinical information

(overall survival, response to immunotherapy, etc.) for 105 melanoma patients with a cutaneous primary and who were treated with programmed cell death protein 1 (PD-1) blockade. In this dataset, response to tumor immunotherapy was defined according to the Response Evaluation Criteria in Solid Tumors 1.1 (RECIST 1.1) criteria. Patients with complete response (CR) or partial response (PR) were considered responders; patients with stable disease(SD) or progressive disease(PD) were considered non-responders. Another dataset, curated by Snyder et al., included 44 primary cutaneous melanoma patients who received T-lymphocyte-associate antigen 4(CTLA-4) blockade therapy. Unlike the dataset presented by Liu et al., this cohort defined patients with long benefit (LB) as responders and patients with non-benefit (NB) as non-responders. Detailed information on all cohorts used in this study is provided in Supplementary Tables S1 and S2.

We downloaded 323 pathways from the Kyoto Encyclopedia of Genes and Genomes (KEGG) database (https://www.genome.jp/kegg/) [30, 31], encompassing metabolism, membrane transport, signal transduction, cell cycle, etc. The human-specific PPI network was obtained from 12 sources collected by previous researchers [32, 33]. To obtain high-confidence links, we further filtered PPIs from more than two sources. This measure for filtering PPIs has been used in previous studies [13]. Finally, the largest connected sub-network containing 12436 nodes and 83020 edges was extracted using the “igraph” package.

### The ssMutPA framework

ssMutPA was developed to calculate the mutation-based individualized pathway activity profiles. To do this, we mapped the somatic mutations to the genes in the PPI network as seed nodes for each individual sample. Then, the local and global weighted strategies were used to calculate the influenced scores of genes based on the mutation constraints imposed by the network topology. The local weighted strategy considered the mutation frequency of the

neighbors of seed nodes, whereas the global weighted strategy used an iterative propagation algorithm to evaluate the extent of genes influenced by mutation genes along the network. Ultimately, we calculated a mutation-based pathway enrichment score to reflect the accumulative effect of mutation genes on each pathway.

**Local weighted strategy**

A mutation gene with a high degree in the PPI network may play critical functional roles [34], which may be reinforced by its neighbor mutations. Considering the number of mutation genes in the neighbors of each seed node, we proposed a local weighted strategy to distinguish the importance of seed nodes for each sample. Specifically, suppose that the background network has a total of $N$ genes, of which $K$ are seed nodes under investigation in the sample. For a given seed node ($G_i$), there are two variables to characterize if any $G_i$ may be reinforced by the mutations of its neighbors: the number of mutation genes in the neighbors of $G_i$, designated as $X_i$, and the number of neighbors of $G_i$, designated as $M_i$. Thus, $X_i$ will follow a hypergeometric distribution and the formula is as follows:

$$p(X_i = x) = \frac{\binom{M_i}{x}\binom{N-M_i}{K-x}}{\binom{N}{K}},\ i = 1,2,\cdots K \qquad (1)$$

For a seed $G_i$ in the sample, if it plays more important function in the disease progression, the number of mutation genes in its neighbors will be significantly larger than the expectation $E(X_i)$, which can be calculated as:

$$E(X_i) = \frac{M_i K}{N} \qquad (2)$$

Thus, we applied a rescaled form of $X_i - E(X_i)$ to quantify the important strength of $G_i$ in the sample, which was defined as local weight $W_i$:

$$w_i = X_i - E(X_i) \qquad (3)$$

$$W_i = log_{\alpha}(w_i I(w_i) + \alpha) \qquad (4)$$

where $I$ is an indicator function, if $w_i$ greater than 0, its value is equal to 1, otherwise equal to 0; and $\alpha$ is a scalar base to guarantee the weight has a minimum value of 1, here we set $\alpha$ as 2.

**Global propagation-based weighted strategy**

Mutation genes can impact not only the activities of their neighboring genes but also other genes in the network due to network topology. We applied a global propagation algorithm, random walk with restart (RWR), to estimate the probable influence of nodes in the network by seed nodes(mutation genes). The RWR algorithm mimics an iterative random walker that, at each time step in the graph, begins from a group of source nodes (here corresponding to mutation genes) and moves either to its immediate neighbors or returns to the source nodes. This algorithm, which captures global relationships within a network, has been effectively used to discriminate disease genes previously [35]. Given that different seed nodes have different important strengths, we improved the RWR algorithm by assigning the initial seed nodes with different weights. The improved RWR model is as follows:

$$p_{t+1} = (1 - c)Ap_t + cp_0 \qquad (5)$$

where $p_0 = (p_0^1, p_0^2, \cdots p_0^N)$ is the initial probability vector, which was constructed by assigning to each seed node with its local weight value and remaining nodes with 0, and then it was normalized to a unit vector that sums to 1. A is the column-normalized adjacency matrix of the PPI network; the parameter $c$ is a certain probability of continuing the random walk or restarting from the restart set, which has been reported to have only a slight effect on the results when it varied between 0.1 and 0.9 [35-37], and we set c=0.7 in the study. $p_t = (p_t^1, p_t^2, \cdots p_t^N)$ is a vector containing visiting probabilities of all nodes in the network at time point $t$. It will

reach a steady state at certain number of iterations, which is obtained when the difference between $p_{t+1}$ and $p_t$ falls below 10e-10. The $p_{t+1}$ reflects the extent to which seed nodes influence other nodes in the network and whose elements are defined as global weights of nodes.

**Calculate single-sample mutation-based pathway enrichment score**

For each sample, we constructed a gene list $L = (g_1, g_2, ... g_N)$ by ranking the genes according to the normalized global weights and mapped the pathways to the ranked gene list respectively. We then calculated a single-sample mutation-based pathway enrichment score (ssMutPES) for each pathway, which reflects how much a pathway is overrepresented at the top of the ranked gene list *L*. The weighted Kolmogorov-Smirnov statistic was used to calculate the ssMutPES. In particular, we calculated the fraction of genes not in the pathway ($F_{NotP}$) and the fraction of genes in the pathway ($F_{InP}$) weighted by their global weights at a given position *i* in the list *L*. and the formulas are as follows:

$$F_{InP}(i) = \sum_{\substack{g_j \in P \\ j \le i}} \frac{|r_j|^p}{N_R} \qquad (6)$$

$$F_{NotP}(i) = \sum_{\substack{g_j \notin P \\ j \le i}} \frac{1}{N_{NotP}} \qquad (7)$$

Where $N_R = \sum_{g_j \in P} |r_j|^p$; $r_j$ is the global weight of gene *j*; $N_{NotP}$ represents the number of genes in the list *L* not in the pathway; *p* controls the extent of gene global weight, we set *p*=1 as default value. The ssMutPES of pathway *P* is determined by going along the list *L* from position *i*:

$$ssMutES = max_{i \in L}(F_{InP}(i) - F_{NotP}(i)) \qquad (8)$$

A pathway with a large ssMutPES value indicates the pathway located at the very top of the

list *L*, suggesting the pathway activity may tend to be induced by the mutation genes. Thus, we refer to the ssMutPES as mutation induced pathway activity hereafter. To prevent any potential confusion, we assigned the ssMutPES of pathway as zero if its ssMutPES<0 which indicates the mutation genes have slight effect on the pathway.

### Identifying cancer subtypes

To assess the performance of the ssMutPA approach in real-world data analysis, we applied it to 33 different cancer types in TCGA. For each cancer type, we first identified the prognostic pathways using the univariate Cox proportional hazards regression model, and then constructed a patient-patient similarity matrix based on the Euclidean distance between pathway activities of samples. The spectral clustering algorithm, which has stronger adaptability to data distribution and excellent clustering effect, was used to classify samples into different subtypes. We applied "specc" function from the "kernlab" package [38] to implement this algorithm. To ascertain the optimal number of clusters, we employed the algorithm of the maximum value of the index, executed by the "Nbclust" function from the "Nbclust" package [39], and we set the "index" parameter as "silhouette", which avoids over-clustering and produces clusters with very small sample size. Moreover, the Kaplan-Meier curve analysis and log-rank test were employed to test the prognostic differences among subtypes.

## Results

### Performance of ssMutPA in cancer stratification

Recently, single-sample pathway (or gene set) enrichment analysis approaches have received extensive attention and promoted the development of precision medicine. However, these approaches mainly focus on gene expression data and do not consider gene mutation information. In the study, we proposed a novel ssMutPA approach that uses network-based

local and global weighted strategies to calculate the ssMutPES, reflecting mutation-based pathway activity. The detailed framework of ssMutPA is shown in Figure 1.

To test whether ssMutPA could effectively identify aberrant pathways associated with clinical prognosis, we applied it to 33 cancer types from the TCGA database. For each cancer, we calculated ssMutPESs of 323 KEGG pathways, and then performed survival analysis using the univariate Cox proportional hazards regression. It was shown that the protective (HR<1) and risk (HR>1) prognostic pathways (cox p-value<0.05) varied among the 33 different cancer types (Figure 2A). Through comparing, we observed that most pathways are associated with only a minimal number of cancers; even some of them are cancer-specific (Figure 2B). Whereas, there are 14 common pathways shared by at least six cancer types, including Mismatch repair, T cell receptor signaling pathway, Regulation of actin cytoskeleton, MAPK signaling pathway, etc. (Figure 2C). Most of these pathways have been reported by previous studies to be associated with the occurrence and progression of diseases. For example, Mismatch repair is involved in DNA replication and gene recombination progress of cells in the human body and is essential for maintaining genome stability, and loss of mismatch repair function leads to microsatellite instability, which may affect disease prognosis or response to drugs [40, 41]. T cells play a pivotal role in the immune response and are part of the adaptive immune system that fights against a variety of infections and cancers [42, 43]. According to the ssMutPESs of these two pathways, we respectively performed Kaplan-Meier curve analysis and log-rank test in their significant associated cancer types. For each pathway, we used the "surv_cutpoint" function in the "survminer" package (https://CRAN.R-project.org/package=survminer) to determine the optimal cut-point of ssMutPESs. The results revealed that the patients in each corresponding cancer type could be classified into two subgroups with significant differences in overall survival (OS) (log-rank test, p<0.05) (Supplementary Figure S1A-B). Moreover, we found that the distributions of ssMutPESs

between high-score and low-score subgroups presented significant differences (Supplementary Figure S1C-D).

To test if the pathway ssMutPESs could stratify cancer patients into clinically relevant subtypes, we performed an unsupervised spectral clustering algorithm on the ssMutPES profiles of prognostic pathways in each of the 33 cancer types. For each cancer type, we used the algorithm of maximum value of the index to determine the relevant number of clusters (see Materials and Methods). We found that each cancer type could be stratified into two to four subtypes, and the subtypes exhibited significant differences in patient prognosis (OS, log-rank test, $p<0.05$) across all cancer types except mesothelioma (MESO) (Figure 3).

**Application of the ssMutPA approach in glioma**

To illustrate the performance of ssMutPA in more detail, we applied it to glioma in TCGA. Glioma is one of the most common primary brain tumors and is usually associated with high morbidity and mortality. In TCGA, gliomas were categorized as Glioblastoma multiforme (GBM) and Brain Lower Grade Glioma (LGG) in accordance with the degree of malignancy; whereas in this study, we merged the two datasets to systematically identify glioma subtypes and performed subsequent analyses. Firstly, we performed univariate Cox proportional hazards regression analysis on the ssMutPES profiles for each pathway in GBM and LGG, and 215 pathways (Supplementary Table S3) associated with overall survival were identified (cox p-value<0.05). According to the ssMutPESs of these pathways, the patients were classified into two subtype clusters (Class 1 and 2) through the spectral clustering algorithm (Supplementary Figure S2), and the top 50 most significant pathways were used to show our results in detail (Figure 4). Through comparing the patients between the subtypes, it was found that the Class 1 subtype primarily consists of GBM patients, while the Class 2 subtype mainly consists of LGG patients. Moreover, we found that the pathways were clustered into four groups (Group

1 to 4). The pathways in Group 2 primarily involved metabolic pathways such as glutathione metabolism, glyoxylate and dicarboxylate metabolism, pentose phosphate pathway, etc., and the ssMutPESs of these pathways in the patients of Class 2 subtype are significant higher than that of the Class 1 subtype patients (Wilcoxon rank-sum test, $p<0.001$). While the pathways in other groups (Group 1, 3, 4) mainly included signaling pathways, and their ssMutPESs are significant higher in the patients of the Class 1 subtype compared with Class 2 subtype. More importantly, the glioma pathway was identified with higher ssMutPESs in the Class 1 subtype patients, which indicates that the Class 1 subtype patients accumulated with more gene mutations in the pathway (Figure 4). These findings illustrated that Class 1 subtype patients were characterized by mutation-induced signaling pathways, whereas Class 2 subtype patients were characterized by mutation-induced metabolic pathways.

To assess the association of subtypes with clinical characteristics, we first performed survival analysis. The result showed that the patients of the Class 2 subtype showed significantly better prognosis than the Class 1 subtype (Kaplan-Meier survival analysis, log-rank test, $p<0.0001$) (Figure 5A). We then compared our subtypes with the clinically relevant glioma subtypes, including pro-neural (PN), Neural (NE), Classical (CL), and Mesenchymal (ME) [44]. Survival analysis demonstrated that the OS of NE/PN subtypes was significantly longer than that of the CL/ME subtypes; however, samples across four different subtypes were not completely separated from each other (Figure 5B). By comparing with our subtypes, we found that the CL/ME patients are mainly included in the Class 1 subtype, while NE/PN patients are mainly included in the Class 2 subtype (Figure 4 and Figure 5C). Moreover, in each original subtype cohort, we respectively performed survival analysis according to our subtypes. Interestingly, the patients in each original subtype could be classified into Class 1 and Class 2 groups (Figure 5D and Supplementary Figure S3). These results suggested that our pathway-based subtypes may complement the original subtypes and promote the development of precision medicine.

Furthermore, we compared the tumor mutation burden (TMB) between Class 1 and Class 2 subtype patients and found that Class 1 subtype patients exhibited significantly larger TMB values than Class 2 (Wilcoxon rank-sum test, $p<2.20e-16$, Figure 5E). This implied that the Class 1 subtype patients were accumulated with more mutations, which resulted in poor prognosis.

Finally, we tested the differences in tumor microenvironment (TME) related characteristics between Class 1 and Class 2 subtypes. Based on the gene expression data of the TCGA glioma patients, we calculated the TME cell infiltration levels according to the cell-type identification by estimating relative subsets of RNA transcripts (CIBERSORT) method [45] and found that macrophages (M0, M1, M2), CD8+T cell, and T follicular helper cells etc. showed significant higher infiltration level in the Class 1 subtype patients than that of Class 2 subtype (Supplementary Figure S4A). We also evaluated the immune score, stromal score, and tumor purity with the ESTIMATE method [46]. Intriguingly, the immune score and stromal score were notably higher in the Class 1 subtype compared with Class 2 subtype (Wilcoxon rank-sum test, $p<0.001$), while the tumor purity exhibited the opposite result (Supplementary Figure S4B-D). The above results indicated that Class 1 subtype patients generally present higher immune activities.

**Identifying pathway-based cancer subtypes associated with response to ICI**

To further test whether the ssMutPA approach could identify key pathways and cancer subtypes associated with response to ICI. We applied ssMutPA to the Liu et al. cohort, comprising 105 melanoma patients treated with the PD-1 inhibitor [28]. According to the ssMutPESs of pathways, 37 survival-related key pathways were identified with the univariate Cox proportional hazards regression analysis ($p<0.05$). Based on the ssMutPESs of these pathways, two subtypes (Class 1 and Class 2) were obtained by using the spectral clustering algorithm.

We found that the Class 1 subtype patients presented a longer OS (log-rank test, p=2.30e-04, Figure 6A) and a higher objective response rate (ORR) than the Class 2 subtype patients (Fisher's exact test, p=3.26e-03, Figure 6B). Among the identified key pathways, several were immune-related, such as the T cell receptor signaling pathway and cellular senescence, etc. We then detected the mutation patterns of the top 20 genes in terms of mutation rate within the T cell receptor signaling pathway. The result showed that the mutation rates of these genes in Class 1 subtype patients were obviously higher than Class 2 subtype (Supplementary Figure S5A). Additionally, we compared TMB between Class 1 and Class 2 subtypes and found that the patients of Class 1 subtype present higher TMB values than Class 2 subtype (Wilcoxon rank-sum test, p<2.60e-05, Figure 6C). Moreover, we applied the ssMutPA approach to the Snyder et al. cohort, comprising 44 melanoma patients treated with CTLA-4 [29]. Following the same analysis as described above, nine key pathways associated with OS were found and the melanoma patients were also clustered into two distinct subtypes. Consistently, the patients of Class 1 subtype presented a longer overall survival (log-rank test, p<0.0001, Figure 6D) and a higher ORR than the patients of Class 2 subtype (Fisher's exact test, p=3.45e-03, Figure 6E). Investigating the key pathways, some important pathways such as the IL-17 signaling pathway and ECM-receptor interaction, were identified and which have been reported to be frequently activated or mutated in cancer. By comparing the mutation status of the top 20 genes in the IL-17 signaling pathway between Class 1 and Class 2 subtypes, we found that these genes are more frequently mutated in Class 1 subtype (Supplementary Figure S5B). Finally, we also found that Class 1 subtype patients showed higher TMB than Class 2 subtype (Wilcoxon rank-sum test, p<6.60e-06, Figure 6F). These results illustrated that the ssMutPA approach could effectively identify mutation-induced aberrant pathways and cluster melanoma patients into subtypes with different prognoses and immunotherapy responses.

## Comparison of ssMutPA with other individualized pathway activity analysis methods based on transcriptomic data

To explain whether the ssMutPA approach could provide new biological insights, we compared it with other individualized pathway activity analysis methods, including GSVA, ssGSEA, iPath, and Pathifier. As some of these methods require normal samples to infer pathway activities, we used 14 cancer types from TCGA (BLCA, BRCA, COAD, etc.), each of which includes at least 20 normal samples. For a fair comparison, we examined the prognostic prediction performance of each method. Specifically, we respectively applied these methods to each cancer dataset to obtain individualized pathway activity profiles. We then used the forward-stepwise algorithm to determine the optimal prognostic pathway sets with the highest predictive power (the concordance index, C-index, was used) and constructed a pathway-based prognostic signature according to the multivariate Cox proportional hazards regression model. Comparing the pathway-based prognostic signatures of each method, we found that the C-index of ssMutPA approach was greater than or equal to that of the other methods across most of the 14 cancer types (Figure 7A). Additionally, we performed time-dependent Receiver Operating Characteristic (ROC) curve analysis for 1-5 years for each method's signature. The results showed that the values of area under the ROC curve (AUROC) of the ssMutPA signature exceeded 0.75 in almost all cancers, which were also comparable to the signatures of other methods (Figure 7B). These results illustrated that the signature of ssMutPA could effectively predict the prognosis of cancer patients. More importantly, the ssMutPA method uses gene mutation data to calculate the pathway activities (ssMutPES), distinct from other methods that use gene expression data. Therefore, the ssMutPA method may provide some new insight into inferring individualized pathway activity and complement the current methods focused on gene expression data.

## Comparison of ssMutPA with non-weighted ssMutPA

Considering the effect of mutation genes may be reinforced by their neighbors in the PPI network, we proposed a novel local weighted strategy to distinguish the importance of mutation genes for each sample. To assess if the local weighted strategy increases the performance of ssMutPA, we compared the clustering results of ssMutPA with that of ssMutPA without local weight (expressed as non-weighted ssMutPA) across 33 cancers in TCGA. Specifically, we applied non-weighted ssMutPA to each cancer type and obtained the ssMutPES profiles of pathways. Subsequently, the same procedure was performed on the ssMutPES profiles to identify cancer subtypes. By comparing the prognostic difference among subtypes (Kaplan-Meier survival analysis), we found that the performance (p-value of the log-rank test) of ssMutPA outperformed non-weighted ssMutPA in almost all cancer types (Figure 7C). This indicated that the local weighted strategy is essential to ssMutPA, which increases its predicted efficacy.

## Discussion

Currently, an increasing number of single-sample pathway activity calculation methods and tools are being developed for identifying dysregulated pathways in complex diseases [14-17]. However, almost all methods focus on gene expression data, overlooking the gene mutation data because of its sparseness and discreteness. In this study, ssMutPA was developed to infer individualized pathway activities by integrating somatic mutation data and PPI network topology. To demonstrate the effectiveness of ssMutPA, we applied it to 33 cancer types from the TCGA database. Based on the mutation-induced pathway activity (ssMutPES) profiles, the patients could be clustered into different subtypes with significantly different prognoses in each cancer type. When comparing the ssMutPA approach with other individualized pathway activity analysis methods, including GSVA, ssGSEA, iPath, and Pathifier, we found that the prognosis prediction power of ssMutPA-based signature was superior to other methods. This

indicated that the mutation-based individualized pathway analysis may complement the existing methods focused on gene expression data and provide some new insights into cancer precision medicine.

Because of the sparseness and discreteness of mutations, we mapped them to the PPI network to evaluate the effect of mutations on network genes. As the different mutation genes generally possess different network topology, they may perform different influences on diseases. We thus proposed a novel local weighted strategy, which takes into account the difference in network topology and the number of mutated genes in the neighbor of each seed node, to determine the importance of mutation genes in every sample (see Method). This strategy not only emphasizes the importance of mutated genes themselves but also indicates the degree to which the mutated genes are affected by neighbor nodes in the network. It is particularly meaningful for our ssMutPA method. To demonstrate the importance of the local weight, we compared the ssMutPA method with the method without local weight (defined as non-weighted ssMutPA), and the results showed that the ssMutPA method was superior to the non-weighted ssMutPA method in the clustering performance (Figure 7C). This indicated that the local weighted can improve the performance of method and is crucial for the ssMutPA method.

In summary, this study presents a novel ssMutPA method for inferring individualized pathway activities by integrating somatic mutation data and the PPI network. The mutation-based individualized pathway activity profiles could effectively reveal patient stratification with significantly different prognoses. Moreover, the ssMutPA outperformed the current individualized pathway analysis methods focused on gene expression data in prognostic prediction performance and thus may complement these methods. Finally, we implemented ssMutPA as an R-based software package, which is available at https://CRAN.R-project.org/package=ssMutPA.

## Availability of Source Code and Requirements

Project name: ssMutPA

Project homepage: https://CRAN.R-project.org/package=ssMutPA

Operating system(s): Platform independent

Programming language: R 3.5.0 or higher

Other requirements: R packages ggplot2, ggridges, grDevices, igraph, kernlab, maftools, Matrix, NbClust, parallel, pheatmap, RColorBrewer, stats, survival, utils.

License: GPL 2.0 or higher

## Data Availability

The details of the patient cohorts used for pan-cancer analysis and case studies in this study are listed in Supplementary Tables S1 and S2. The pathways used for ssMutPA are obtained from the KEGG database. The integrated PPI network and the core code implemented for ssMutPA are included in the R package ssMutPA, which is freely available on CRAN (https://CRAN.R-project.org/package=ssMutPA).

## Additional Files

**Supplementary Figure S1.** Individual pathway prognostic analysis.

**Supplementary Figure S2.** Heatmap of the characteristic pathways in glioma. The Sankey diagram above the heatmap displays the correspondence between transcriptome subtypes and the subtypes we identified.

**Supplementary Figure S3.** Further stratification of clinically relevant subtype patients based on ssMutPA-determined subtypes. Kaplan-Meier survival curves of OS comparing the Class 1 and Class 2 patients within clinically relevant subtypes (CL, NE, PN, ME).

**Supplementary Figure S4.** The relationship between glioma subtypes and immune.

**Supplementary Figure S5.** Mutation analysis of genes involved in the characteristic pathways.

**Supplementary Table S1.** The information of all the cohorts we used in this study.

**Supplementary Table S2.** Detailed information on 33 cancer types in the TCGA database.

**Supplementary Table S3.** Prognostically relevant pathway identified in gliomas (cox p-value<0.05).

## Abbreviations

ssMutPA: Single-sample mutation-based pathway analysis; PPI: Protein-protein interaction; ssMutPES: Single-sample mutation-based pathway enrichment score; PEA: Pathway enrichment analysis; GSEA: Gene Set Enrichment Analysis; SPIA: Signaling Pathway Impact Analysis; ssGSEA: single-sample Gene Set Enrichment Analysis; GSVA: Gene Set Variation Analysis; ICIs: Immune checkpoint inhibitors; TCGA: The Cancer Genome Atlas; MAF: Mutation Annotation Format; CR: Complete response; PR: Partial response; SD:Stable disease; PD: Progressive disease; PD-1: Programmed cell death protein 1; CTLA-4: T-lymphocyte-associate antigen 4; LB: Long benefit; NB: Non-benefit; KEGG: Kyoto Encyclopedia of Genes and Genomes; RWR: Random walk with restart; OS: Overall survival; MESO: Mesothelioma; GBM: Glioblastoma multiforme; LGG: Brain Lower Grade Glioma; PN: Pro-neural; NE: Neural; CL: Classical; ME: Mesenchymal; TMB: Tumor mutation burden; TME: Tumor microenvironment; CIBERSORT: Cell-type identification by

estimating relative subsets of RNA transcripts; ORR: Objective response rate; C-index: Concordance index; ROC: Receiver Operating Characteristic; AUROC: Area under the ROC curve.

## Competing Interests

The authors declare that they have no competing interests.


## Funding

National Natural Science Foundation of China (grant no.62072145 and 62372143), the Natural Science Foundation of Heilongjiang Province (grant no. LH2019C042).


## Authors' Contribution

Y.H. and J.H. jointly developed the initial concepts and framework of the study. Q.W. and J.L. assisted in refining the methodology and study design. Y.H. and Q.W. were responsible for the development of the software package. Q.W. and J.L. assessed and confirmed the validity of the study's findings. B.P., S.L., and X.Z. designed and prepared the figures and table. Y.H. was responsible for the initial drafting of the manuscript. J.H. provided the review and extensive editing of the manuscript. All authors read and approved the final manuscript.


## Acknowledgments

Not applicable


## REFERENCES


1. Cantini L, Calzone L, Martignetti L, Rydenfelt M, Bluthgen N, Barillot E, et al. Classification of gene signatures for their information value and functional redundancy. NPJ Syst Biol Appl. 2018;4:2. doi:10.1038/s41540-017-0038-8.
2. Dang H, Pomyen Y, Martin SP, Dominguez DA, Yim SY, Lee JS, et al. NELFE-Dependent MYC Signature Identifies a Unique Cancer Subtype in Hepatocellular Carcinoma. Sci

Rep. 2019;9 1:3369. doi:10.1038/s41598-019-39727-9.
3. Xu Q, Chen J, Ni S, Tan C, Xu M, Dong L, et al. Pan-cancer transcriptome analysis reveals a gene expression signature for the identification of tumor tissue origin. Mod Pathol. 2016;29 6:546-56. doi:10.1038/modpathol.2016.60.
4. Zuo S, Zhang X and Wang L. A RNA sequencing-based six-gene signature for survival prediction in patients with glioblastoma. Sci Rep. 2019;9 1:2615. doi:10.1038/s41598-019-39273-4.
5. Siva N. New gene biomarker identified for indolent prostate cancer. Lancet Oncol. 2013;14 11:e446. doi:10.1016/S1470-2045(13)70431-5.
6. Vargas AJ and Harris CC. Biomarker development in the precision medicine era: lung cancer as a case study. Nat Rev Cancer. 2016;16 8:525-37. doi:10.1038/nrc.2016.56.
7. Diamandis EP. Cancer biomarkers: can we turn recent failures into success? J Natl Cancer Inst. 2010;102 19:1462-7. doi:10.1093/jnci/djq306.
8. Boutros PC. The path to routine use of genomic biomarkers in the cancer clinic. Genome Res. 2015;25 10:1508-13. doi:10.1101/gr.191114.115.
9. Rosario SR, Long MD, Affronti HC, Rowsam AM, Eng KH and Smiraglia DJ. Pan-cancer analysis of transcriptional metabolic dysregulation using The Cancer Genome Atlas. Nat Commun. 2018;9 1:5330. doi:10.1038/s41467-018-07232-8.
10. Ke X, Wu H, Chen YX, Guo Y, Yao S, Guo MR, et al. Individualized pathway activity algorithm identifies oncogenic pathways in pan-cancer analysis. EBioMedicine. 2022;79:104014. doi:10.1016/j.ebiom.2022.104014.
11. Subramanian A, Tamayo P, Mootha VK, Mukherjee S, Ebert BL, Gillette MA, et al. Gene set enrichment analysis: a knowledge-based approach for interpreting genome-wide expression profiles. Proc Natl Acad Sci U S A. 2005;102 43:15545-50. doi:10.1073/pnas.0506580102.
12. Tarca AL, Draghici S, Khatri P, Hassan SS, Mittal P, Kim JS, et al. A novel signaling pathway impact analysis. Bioinformatics. 2009;25 1:75-82. doi:10.1093/bioinformatics/btn577.
13. Liu H, Yuan M, Mitra R, Zhou X, Long M, Lei W, et al. CTpathway: a CrossTalk-based pathway enrichment analysis method for cancer research. Genome Med. 2022;14 1:118. doi:10.1186/s13073-022-01119-6.
14. Barbie DA, Tamayo P, Boehm JS, Kim SY, Moody SE, Dunn IF, et al. Systematic RNA interference reveals that oncogenic KRAS-driven cancers require TBK1. Nature. 2009;462 7269:108-12. doi:10.1038/nature08460.
15. Hanzelmann S, Castelo R and Guinney J. GSVA: gene set variation analysis for microarray and RNA-seq data. BMC Bioinformatics. 2013;14:7. doi:10.1186/1471-2105-14-7.
16. Su K, Yu Q, Shen R, Sun SY, Moreno CS, Li X, et al. Pan-cancer analysis of pathway-based gene expression pattern at the individual level reveals biomarkers of clinical prognosis. Cell Rep Methods. 2021;1 4 doi:10.1016/j.crmeth.2021.100050.
17. Pian C, He M and Chen Y. Pathway-Based Personalized Analysis of Pan-Cancer Transcriptomic Data. Biomedicines. 2021;9 11 doi:10.3390/biomedicines9111502.
18. Chen C, Grennan K, Badner J, Zhang D, Gershon E, Jin L, et al. Removing batch effects in analysis of expression microarray data: an evaluation of six batch adjustment methods. PLoS One. 2011;6 2:e17238. doi:10.1371/journal.pone.0017238.
19. Leek JT, Scharpf RB, Bravo HC, Simcha D, Langmead B, Johnson WE, et al. Tackling the widespread and critical impact of batch effects in high-throughput data. Nat Rev Genet. 2010;11 10:733-9. doi:10.1038/nrg2825.
20. Yaacov A, Rosenberg S and Simon I. Mutational signatures association with replication timing in normal cells reveals similarities and differences with matched cancer tissues. Sci Rep. 2023;13 1:7833. doi:10.1038/s41598-023-34631-9.
21. Nam AS, Kim KT, Chaligne R, Izzo F, Ang C, Taylor J, et al. Somatic mutations and cell identity linked by Genotyping of Transcriptomes. Nature. 2019;571 7765:355-60.

doi:10.1038/s41586-019-1367-0.
22. Li X, He Y, Wu J, Qiu J, Li J, Wang Q, et al. A novel pathway mutation perturbation score predicts the clinical outcomes of immunotherapy. Brief Bioinform. 2022;23 5 doi:10.1093/bib/bbac360.
23. Long J, Wang D, Wang A, Chen P, Lin Y, Bian J, et al. A mutation-based gene set predicts survival benefit after immunotherapy across multiple cancers and reveals the immune response landscape. Genome Med. 2022;14 1:20. doi:10.1186/s13073-022-01024-y.
24. Wang Q, Li X, Qiu J, He Y, Wu J, Li J, et al. A pathway-based mutation signature to predict the clinical outcomes and response to CTLA-4 inhibitors in melanoma. Comput Struct Biotechnol J. 2023;21:2536-46. doi:10.1016/j.csbj.2023.04.004.
25. Jiao X, Wei X, Li S, Liu C, Chen H, Gong J, et al. A genomic mutation signature predicts the clinical outcomes of immunotherapy and characterizes immunophenotypes in gastrointestinal cancer. NPJ Precis Oncol. 2021;5 1:36. doi:10.1038/s41698-021-00172-5.
26. Pan D, Hu AY, Antonia SJ and Li CY. A Gene Mutation Signature Predicting Immunotherapy Benefits in Patients With NSCLC. J Thorac Oncol. 2021;16 3:419-27. doi:10.1016/j.jtho.2020.11.021.
27. Bailey MH, Tokheim C, Porta-Pardo E, Sengupta S, Bertrand D, Weerasinghe A, et al. Comprehensive Characterization of Cancer Driver Genes and Mutations. Cell. 2018;173 2:371-85 e18. doi:10.1016/j.cell.2018.02.060.
28. Liu D, Schilling B, Liu D, Sucker A, Livingstone E, Jerby-Arnon L, et al. Integrative molecular and clinical modeling of clinical outcomes to PD1 blockade in patients with metastatic melanoma. Nat Med. 2019;25 12:1916-27. doi:10.1038/s41591-019-0654-5.
29. Snyder A, Makarov V, Merghoub T, Yuan J, Zaretsky JM, Desrichard A, et al. Genetic basis for clinical response to CTLA-4 blockade in melanoma. N Engl J Med. 2014;371 23:2189-99. doi:10.1056/NEJMoa1406498.
30. Kanehisa M, Sato Y, Kawashima M, Furumichi M and Tanabe M. KEGG as a reference resource for gene and protein annotation. Nucleic Acids Res. 2016;44 D1:D457-62. doi:10.1093/nar/gkv1070.
31. Kanehisa M, Araki M, Goto S, Hattori M, Hirakawa M, Itoh M, et al. KEGG for linking genomes to life and the environment. Nucleic Acids Res. 2008;36 Database issue:D480-4. doi:10.1093/nar/gkm882.
32. Cheng F, Desai RJ, Handy DE, Wang R, Schneeweiss S, Barabasi AL, et al. Network-based approach to prediction and population-based validation of in silico drug repurposing. Nat Commun. 2018;9 1:2691. doi:10.1038/s41467-018-05116-5.
33. Cheng F, Kovacs IA and Barabasi AL. Network-based prediction of drug combinations. Nat Commun. 2019;10 1:1197. doi:10.1038/s41467-019-09186-x.
34. Cheng F, Zhao J, Wang Y, Lu W, Liu Z, Zhou Y, et al. Comprehensive characterization of protein-protein interactions perturbed by disease mutations. Nat Genet. 2021;53 3:342-53. doi:10.1038/s41588-020-00774-y.
35. Kohler S, Bauer S, Horn D and Robinson PN. Walking the interactome for prioritization of candidate disease genes. Am J Hum Genet. 2008;82 4:949-58. doi:10.1016/j.ajhg.2008.02.013.
36. Han J, Li C, Yang H, Xu Y, Zhang C, Ma J, et al. A novel dysregulated pathway-identification analysis based on global influence of within-pathway effects and crosstalk between pathways. J R Soc Interface. 2015;12 102:20140937. doi:10.1098/rsif.2014.0937.
37. Di J, Zheng B, Kong Q, Jiang Y, Liu S, Yang Y, et al. Prioritization of candidate cancer drugs based on a drug functional similarity network constructed by integrating pathway activities and drug activities. Mol Oncol. 2019;13 10:2259-77. doi:10.1002/1878-0261.12564.

38. Karatzoglou A, Smola A, Hornik K and Zeileis A. kernlab – An S4 Package for Kernel Methods in R. Journal of Statistical Software. 2004;11 9:1 – 20. doi:10.18637/jss.v011.i09.
39. Charrad M, Ghazzali N, Boiteau V and Niknafs A. NbClust: An R Package for Determining the Relevant Number of Clusters in a Data Set. Journal of Statistical Software. 2014;61 6:1 – 36. doi:10.18637/jss.v061.i06.
40. Dong L, Jiang H, Kang Z and Guan M. Biomarkers for chemotherapy and drug resistance in the mismatch repair pathway. Clin Chim Acta. 2023;544:117338. doi:10.1016/j.cca.2023.117338.
41. Mouw KW, Goldberg MS, Konstantinopoulos PA and D'Andrea AD. DNA Damage and Repair Biomarkers of Immunotherapy Response. Cancer Discov. 2017;7 7:675-93. doi:10.1158/2159-8290.CD-17-0226.
42. Shah K, Al-Haidari A, Sun J and Kazi JU. T cell receptor (TCR) signaling in health and disease. Signal Transduct Target Ther. 2021;6 1:412. doi:10.1038/s41392-021-00823-w.
43. Hwang JR, Byeon Y, Kim D and Park SG. Recent insights of T cell receptor-mediated signaling pathways for T cell activation and development. Exp Mol Med. 2020;52 5:750-61. doi:10.1038/s12276-020-0435-8.
44. Verhaak RG, Hoadley KA, Purdom E, Wang V, Qi Y, Wilkerson MD, et al. Integrated genomic analysis identifies clinically relevant subtypes of glioblastoma characterized by abnormalities in PDGFRA, IDH1, EGFR, and NF1. Cancer Cell. 2010;17 1:98-110. doi:10.1016/j.ccr.2009.12.020.
45. Newman AM, Liu CL, Green MR, Gentles AJ, Feng W, Xu Y, et al. Robust enumeration of cell subsets from tissue expression profiles. Nat Methods. 2015;12 5:453-7. doi:10.1038/nmeth.3337.
46. Yoshihara K, Shahmoradgoli M, Martinez E, Vegesna R, Kim H, Torres-Garcia W, et al. Inferring tumour purity and stromal and immune cell admixture from expression data. Nat Commun. 2013;4:2612. doi:10.1038/ncomms3612.

# Figures legends

**Figure 1.** The workflow of the ssMutPA method.

**Figure 2.** Overview of the prognostic-relevant pathways identified by ssMutPA in pan-cancer. (A) The number of risk/protective pathways identified in 33 cancer types. (B) The number of overlapped prognostic-relevant pathways. (C) Dot plot of univariate HRs and P-values for the overlapped pathways in corresponding cancers. The color indicates the value of the HR, and the circle size represents the significance of P-values.

**Figure 3.** Kaplan-Meier survival curves of OS comparing the subtypes clustered based on the ssMutPES profiles across 33 cancer types from TCGA.

**Figure 4.** Heatmap of the ssMutPESs of top 50 characteristic pathways in glioma. The line plots on the left of the heatmap illustrate the expression level of pathways in different groups within the two subtypes; the Sankey diagram above the heatmap displays the correspondence between clinically relevant subtypes and the subtypes we identified. On the right, we depicted ridge plots of ssMutPESs of pathways to reflect the distribution of these pathways in the two subtypes. Wilcoxon rank-sum test was used to assess the significance of difference between subtypes: "*" represents the P-value < 0.05; "**" represents the P-value < 0.01; "***" represents the P-value < 0.001.

**Figure 5.** Comparison of the subtypes identified based on the ssMutPES profiles with clinically relevant subtypes in glioma. (A) Kaplan-Meier survival curves of OS comparing subtypes identified based on ssMutPES profiles. (B) Kaplan-Meier survival curves of OS comparing clinically relevant subtypes. (C) The proportion of the clinically relevant subtypes in Class 1 and Class 2 subtypes. (D) The p-value of the log-rank test for clustering each clinically relevant subtype of patients using our method. The black dashed line represents the P-value = 0.05. (E) Compare the TMB level between patients in two subtypes.

**Figure 6.** Identify pathway-based cancer subtypes in the immunotherapy datasets. (A) Kaplan-Meier survival curves of OS comparing the Class 1 and Class 2 groups from the Liu cohort. (B) Comparison of the ORR between the Class 1 and Class 2 groups from the Liu cohort. (C) Comparison of the TMB level between the Class 1 and Class 2 groups from the Liu cohort. (D) Kaplan-Meier survival curves of OS comparing the Class 1 and Class 2 groups from the Snyder cohort. (E) Comparison of the ORR between the Class 1 and Class 2 groups from the Snyder cohort. (F) Comparison of the TMB level between the Class 1 and Class 2 groups from the Snyder cohort.

**Figure 7.** Comparison of ssMutPA with other methods. (A and B) Performance comparison of ssMutPA with other pathway activity algorithms on survival prediction: (A) The C-index of different methods across 14 cancer types; (B) Time-dependent AUC of 1-5 years for different methods across 14 cancer types. (C) Comparing the clustering performance (p-value of the log-rank test) of ssMutPA with non-weighted ssMutPA.

Figure 1

Click here to access/download;Figure;Figure 1.tif

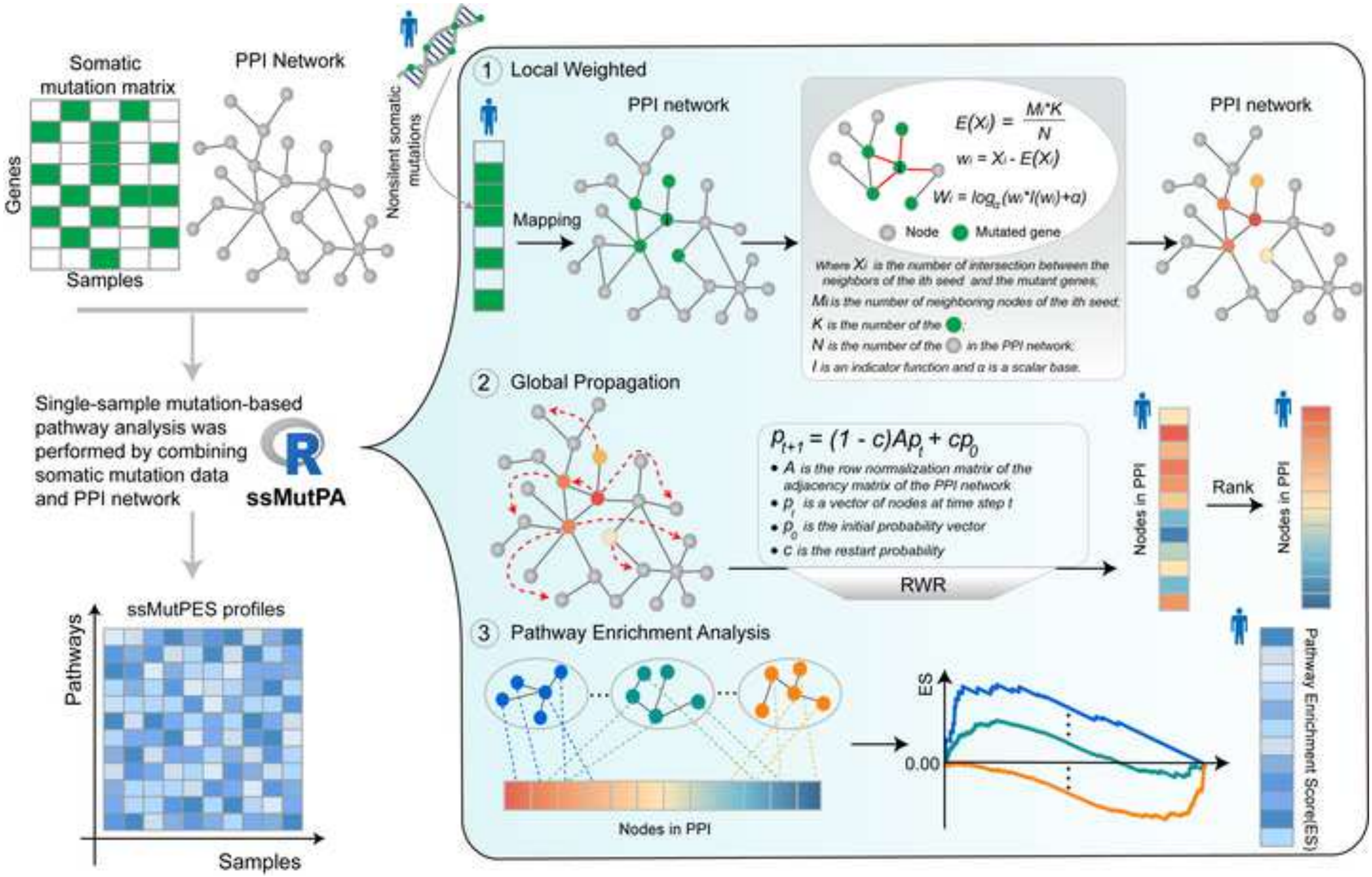

Figure 2

Click here to access/download;Figure;Figure 2.tif

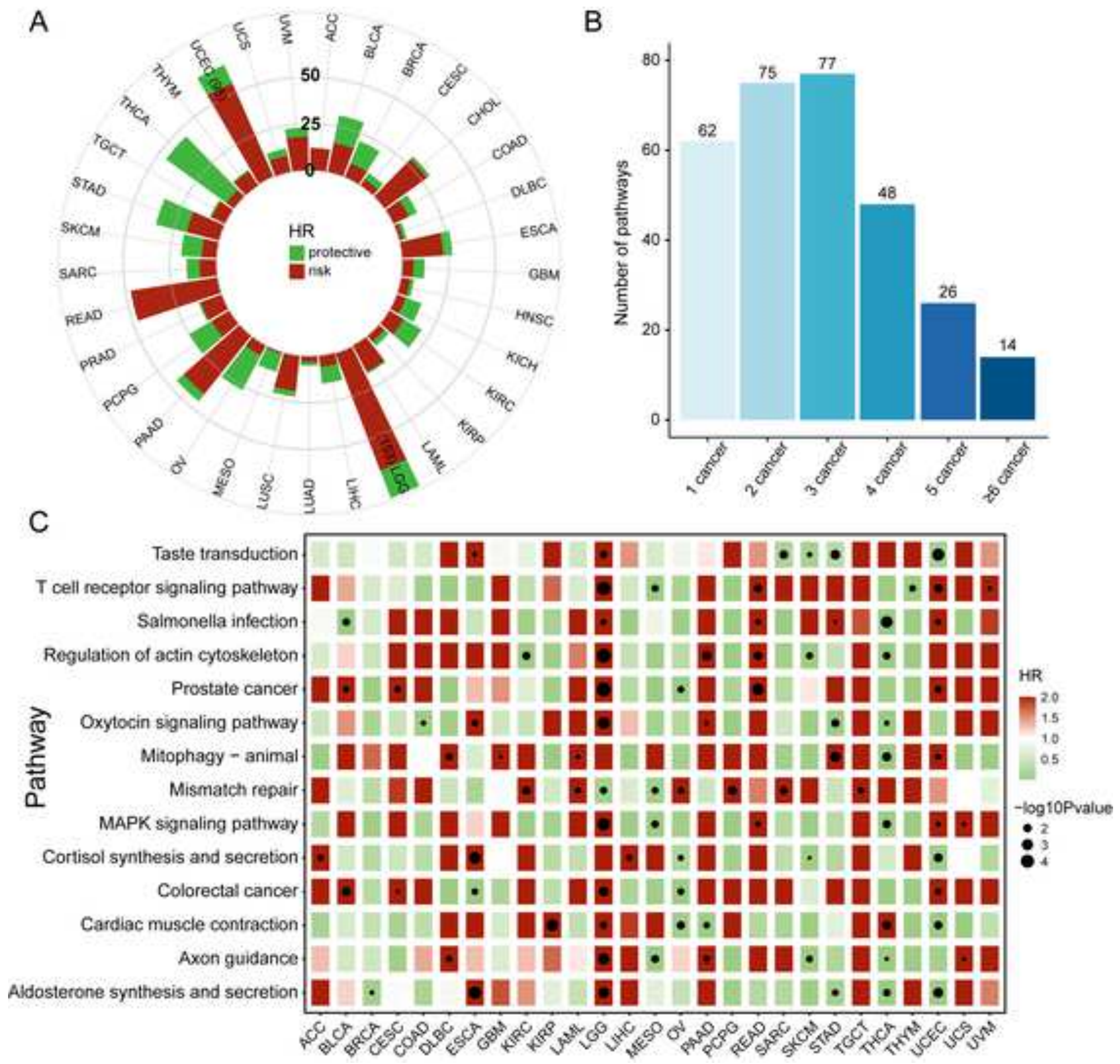

Figure 3 Click here to access/download;Figure;Figure 3.tif

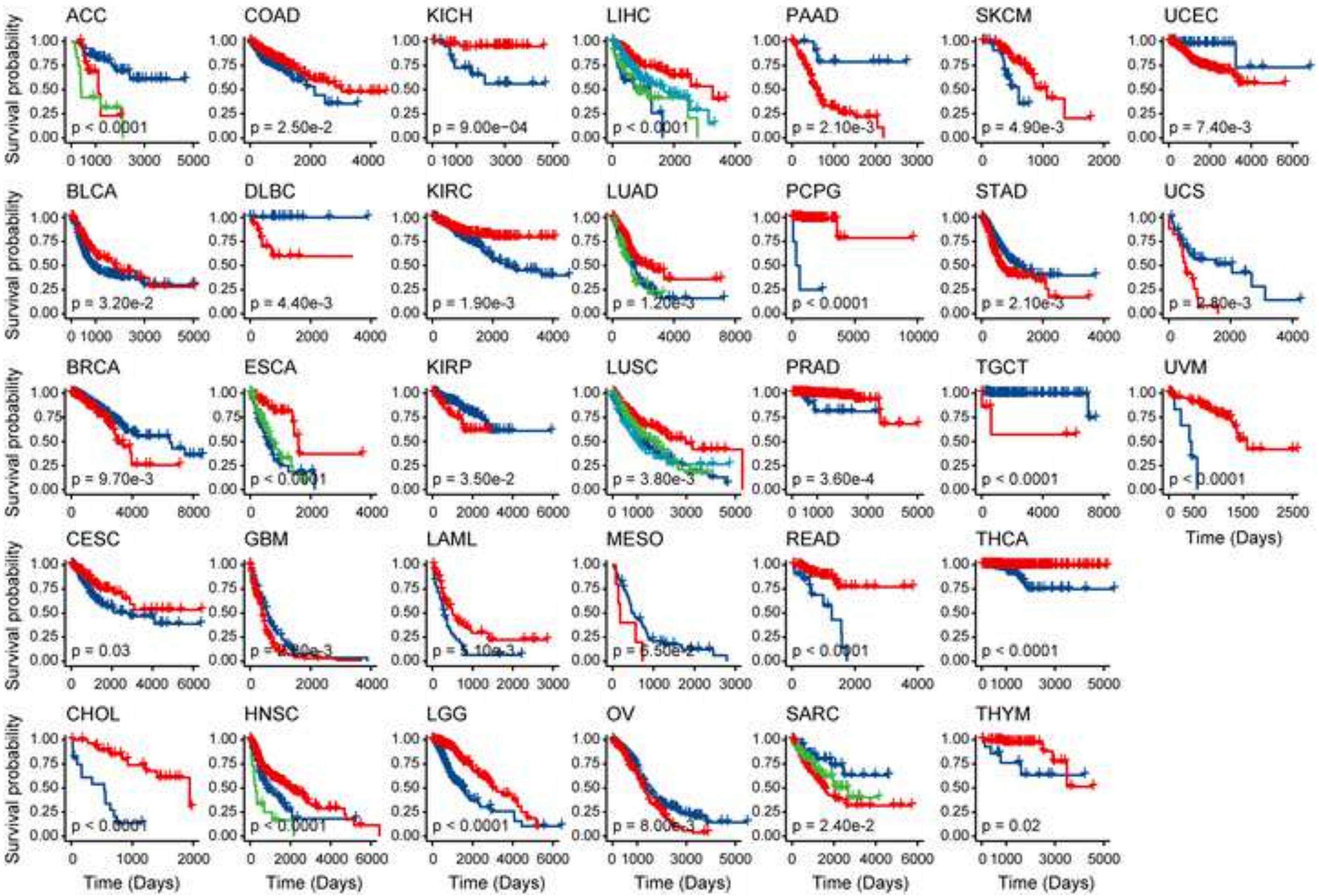

Figure 4

Click here to access/download;Figure;Figure 4.tif

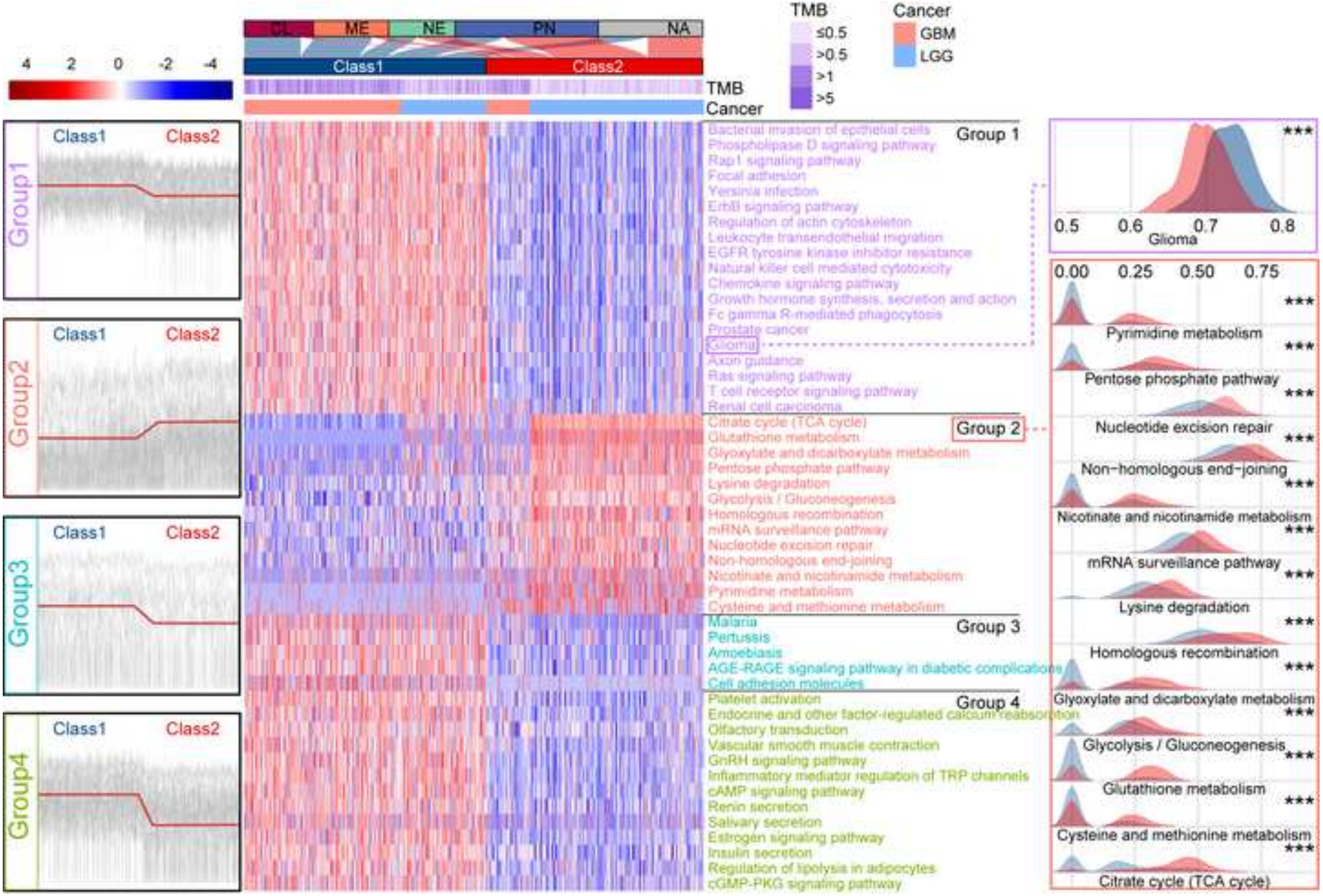

Figure 5

Click here to access/download;Figure;Figure 5.tif

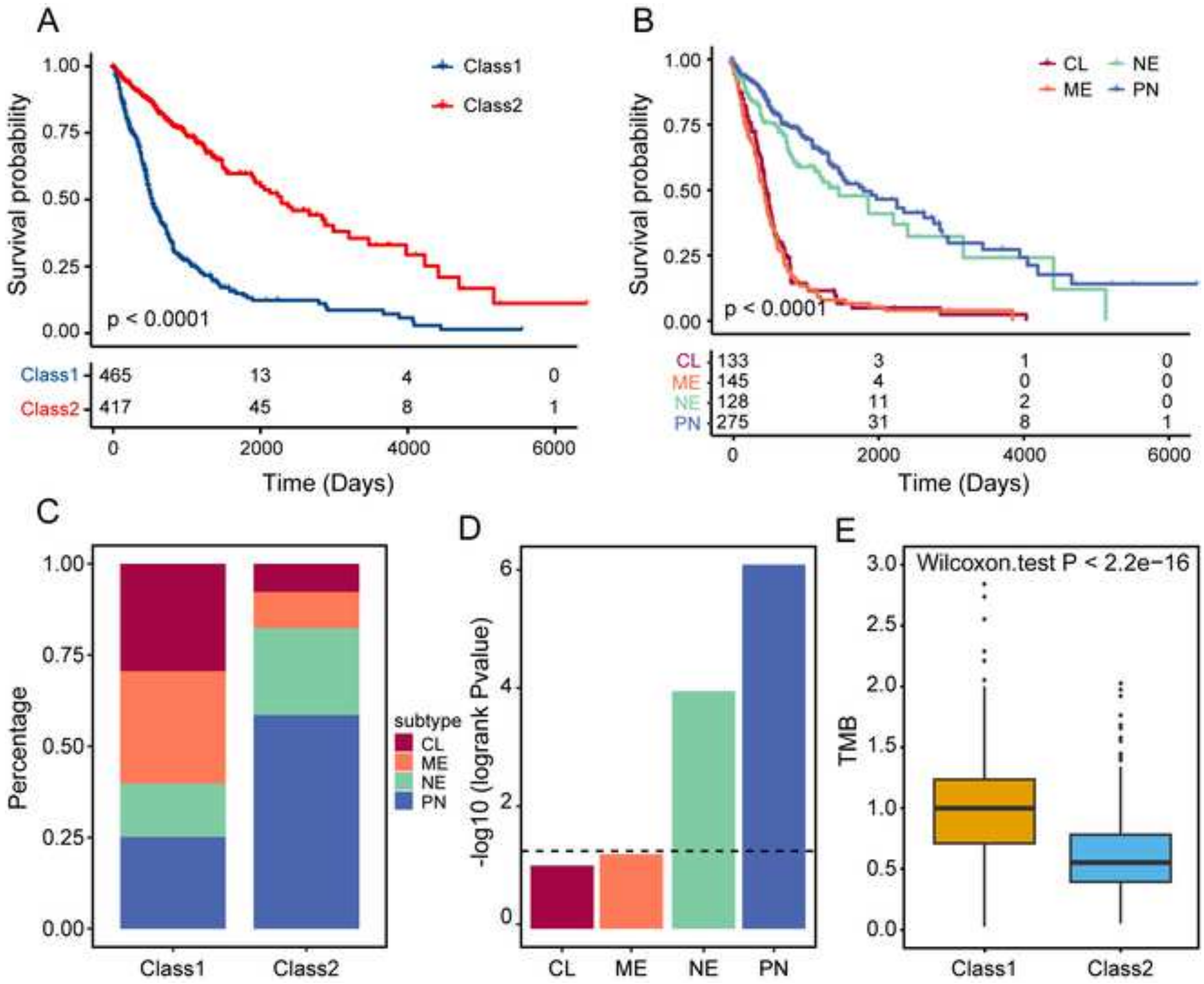

Figure 6

Click here to access/download;Figure;Figure 6.tif

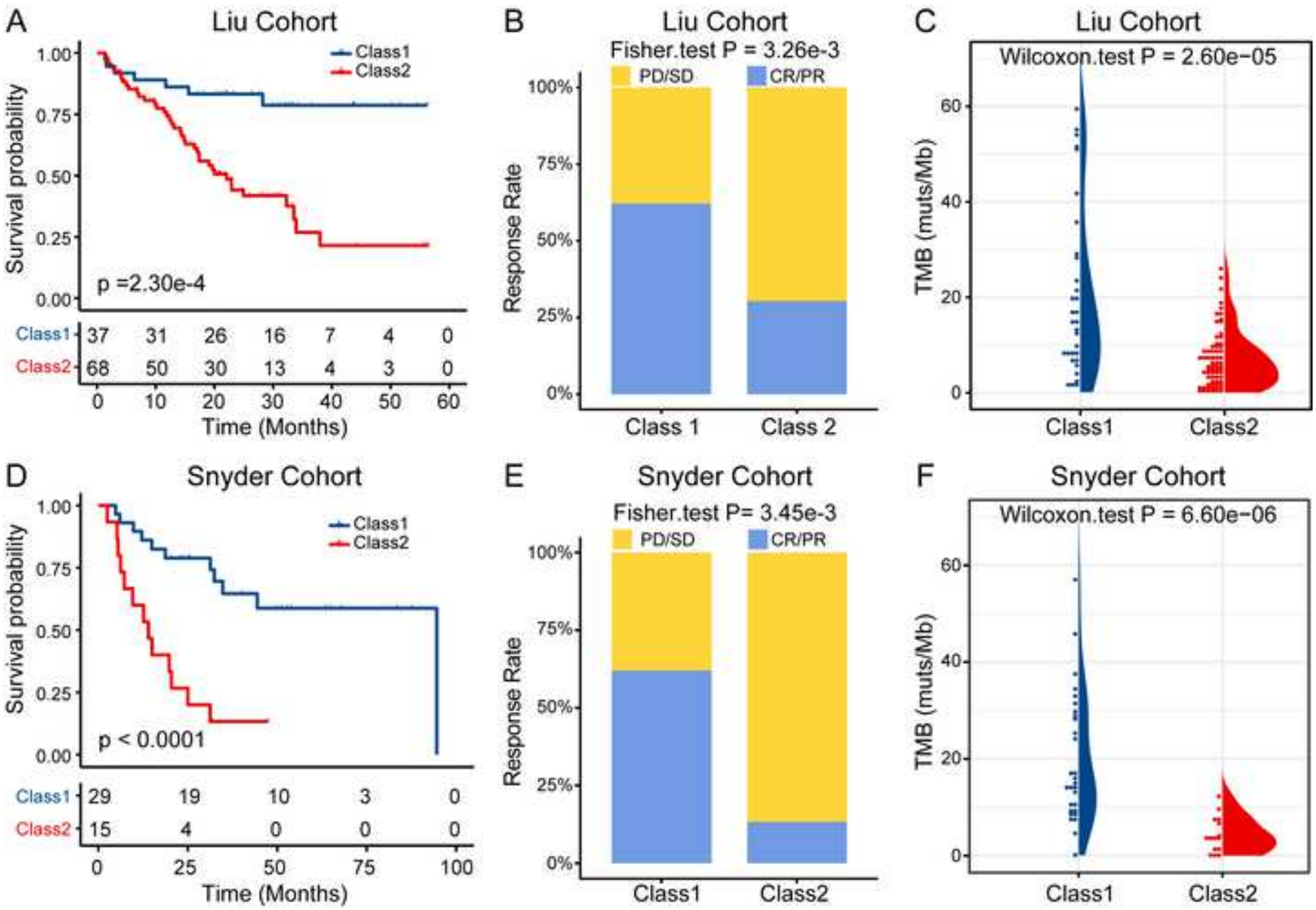

Figure 7

Click here to access/download;Figure;Figure 7.tif

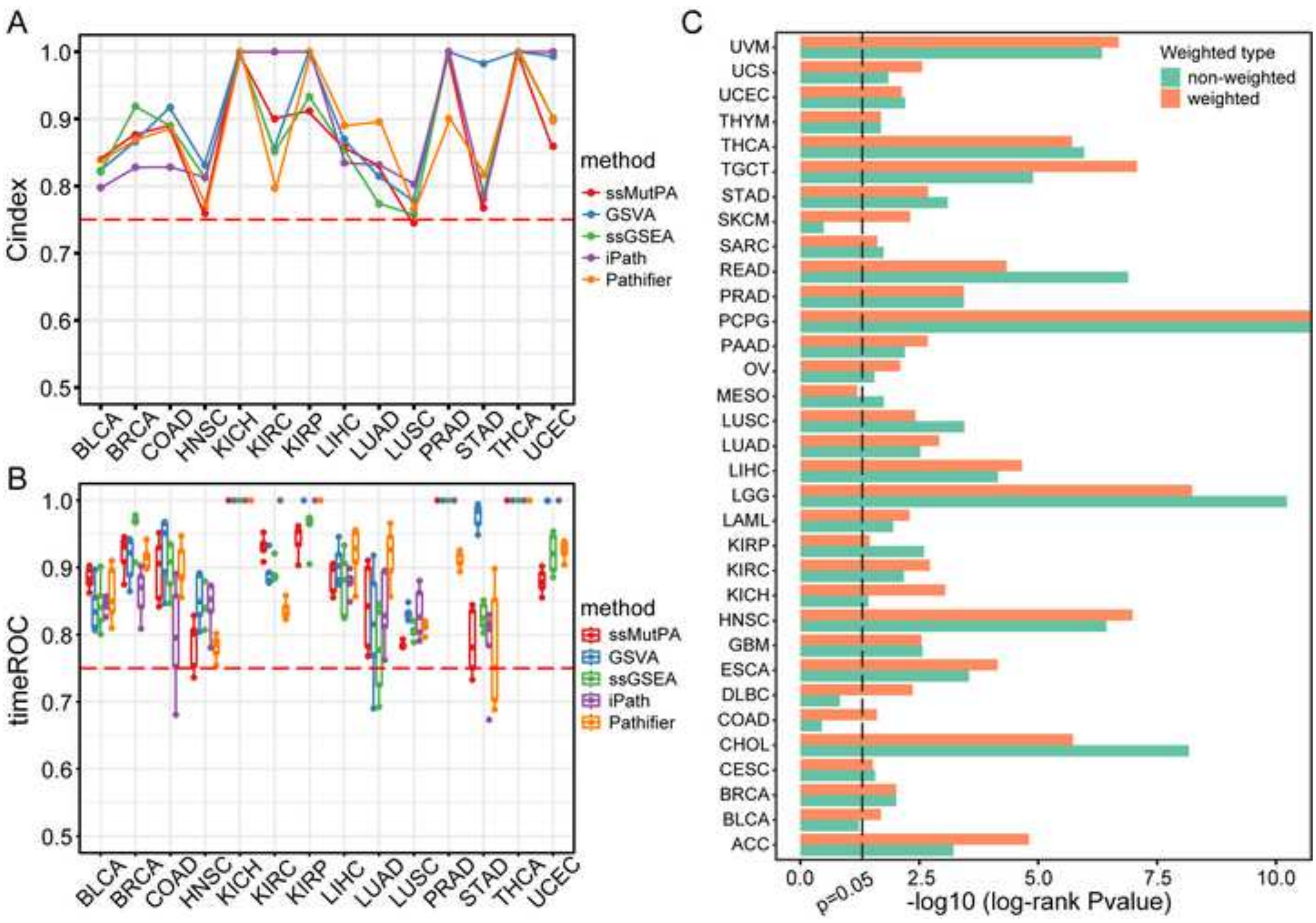

Figure S1_Supplementary Material

Click here to access/download
**Supplementary Material**
Figure S1_Supplementary Material.tif

Figure S2_Supplementary Material

Click here to access/download
**Supplementary Material**
Figure S2_Supplementary Material.tif

Figure S3_Supplementary Material

Click here to access/download
**Supplementary Material**
Figure S3_Supplementary Material.tif

Figure S4_Supplementary Material

Click here to access/download
**Supplementary Material**
Figure S4_Supplementary Material.tif

Figure S5_Supplementary Material

Click here to access/download
**Supplementary Material**
Figure S5_Supplementary Material.tif

Click here to access/download

**Supplementary Material**

Table S1-3_Supplementary Material.xlsx

Click here to access/download
**Supplementary Material**
He Y et al Supplement material.pdf
