# Supplementary material for: ssMutPA: single-sample mutation-based pathway analysis approach for cancer precision medicine
Source: Gigascience. 2024 Dec 20;13:giae105. doi: 10.1093/gigascience/giae105 (PMC11659979; doi:10.1093/gigascience/giae105)
Supplement: giae105_Supplemental_Files [file giae105_supplemental_files.zip › He Y et al Supplement material.pdf]

Supplementary Materials for

ssMutPA: Single-sample Mutation-based Pathway Analysis approach for cancer precision medicine

This PDF file includes:
Supplementary Figure S1-6
Supplementary Table S5

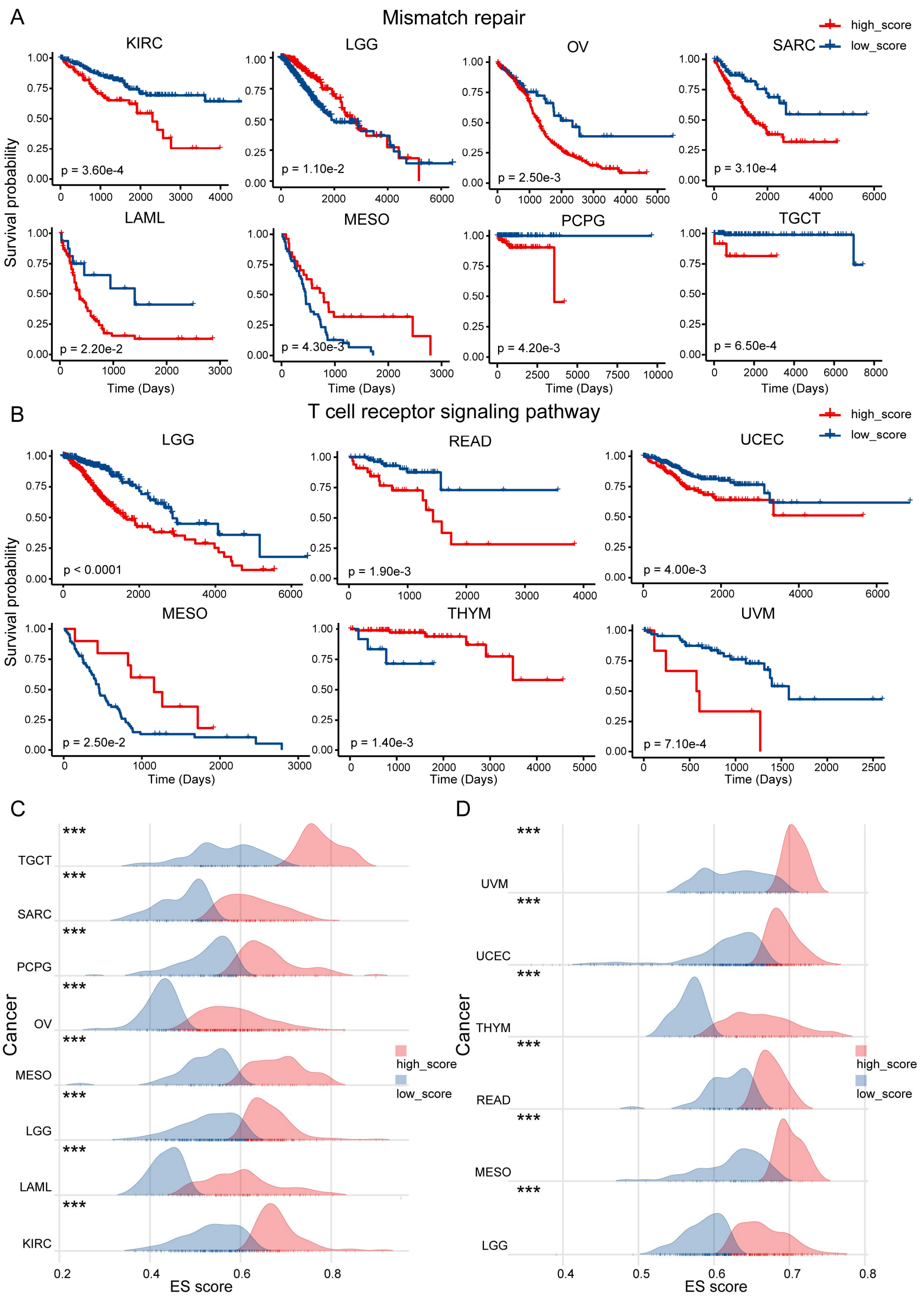

Supplementary Figure S1. Individual pathway prognostic analysis. **(A)** Kaplan-Meier survival curves of OS comparing the high- and low-score groups of Mismatch repair. **(B)** Kaplan-Meier survival curves of OS comparing the high- and low-score groups of T cell receptor signaling pathway. **(C)** Ridgeline plot for comparing the difference of pathway activity between 'high_score' and 'low_score' patient groups of the Mismatch repair pathway. **(D)** Ridgeline plot for comparing the

difference of pathway activity between 'high_score' and 'low_score' patient groups of the T cell receptor signaling pathway. Wilcoxon rank-sum test was used to assess the significance of differences between groups: "*" represents the P-value < 0.05; "**" represents the P-value < 0.01; "***" represents the P-value < 0.001.

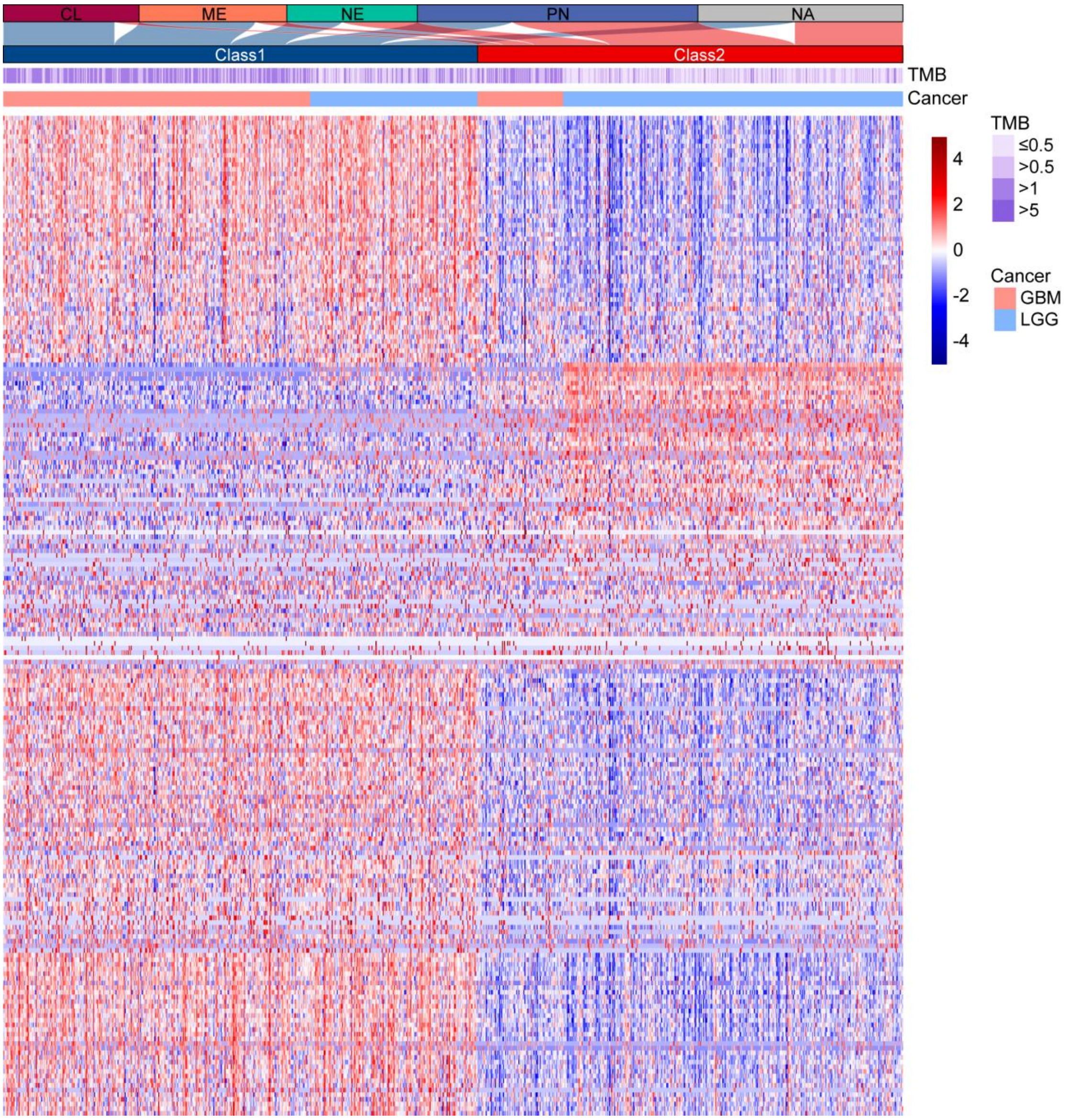

Supplementary Figure S2. Heatmap of the characteristic pathways in glioma. The Sankey diagram above the heatmap displays the correspondence between transcriptome subtypes and the subtypes we identified.

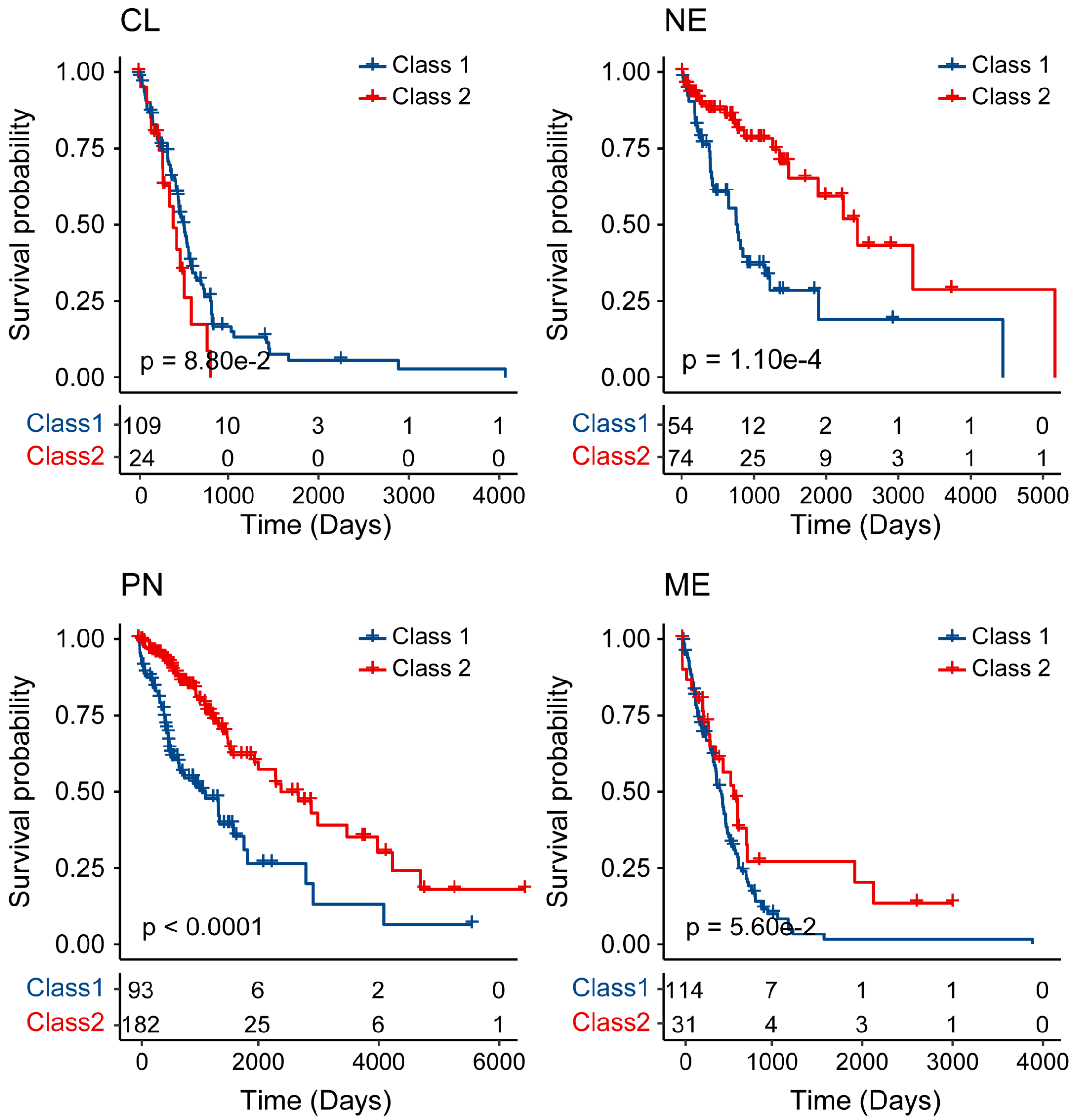

Supplementary Figure S3. Further stratification of clinically relevant subtype patients based on ssMutPA-determined subtypes. Kaplan-Meier survival curves of OS comparing the Class 1 and Class 2 patients within clinically relevant subtypes (CL, NE, PN, ME).

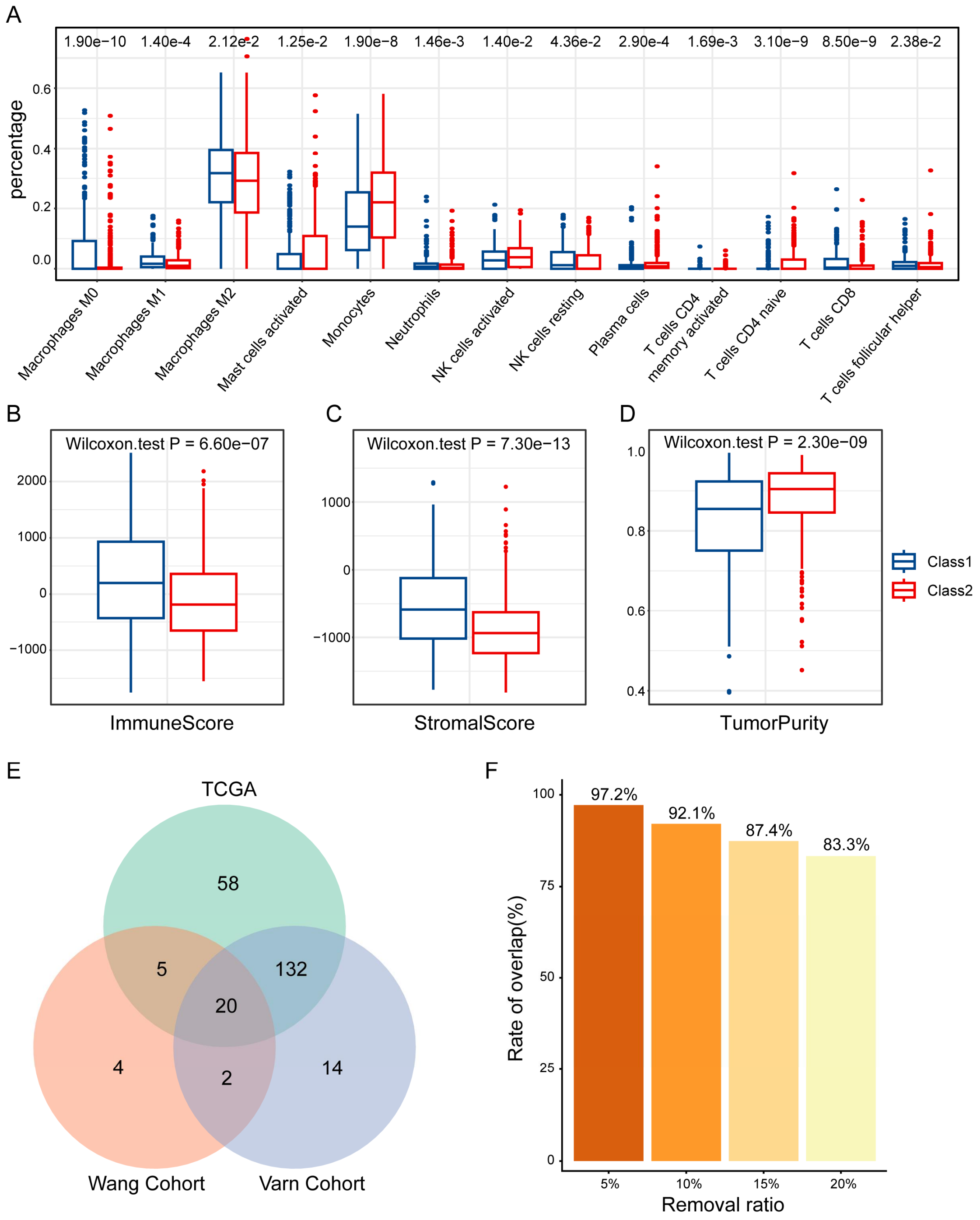

Supplementary Figure S4. Analysis of immune features between glioma subtypes and robustness assessment of the ssMutPA method. **(A)** Box plot of the abundance of significant immune cells between glioma subtypes. The p-value at the top were calculated by the Wilcoxon rank-sum test. **(B-D)** Box plots of stromal score, immune score, and tumor purity between glioma subtypes. **(E)** Venn diagram of the number of overlapped significant pathways identified in the Varn et al. cohort and Wang et al. cohort with those of in TCGA-Glioma cohort. **(F)** Histograms of the overlap number between prognostic pathways identified in TCGA-Glioma after removing different proportions of edges from the network and the original prognostic pathways.

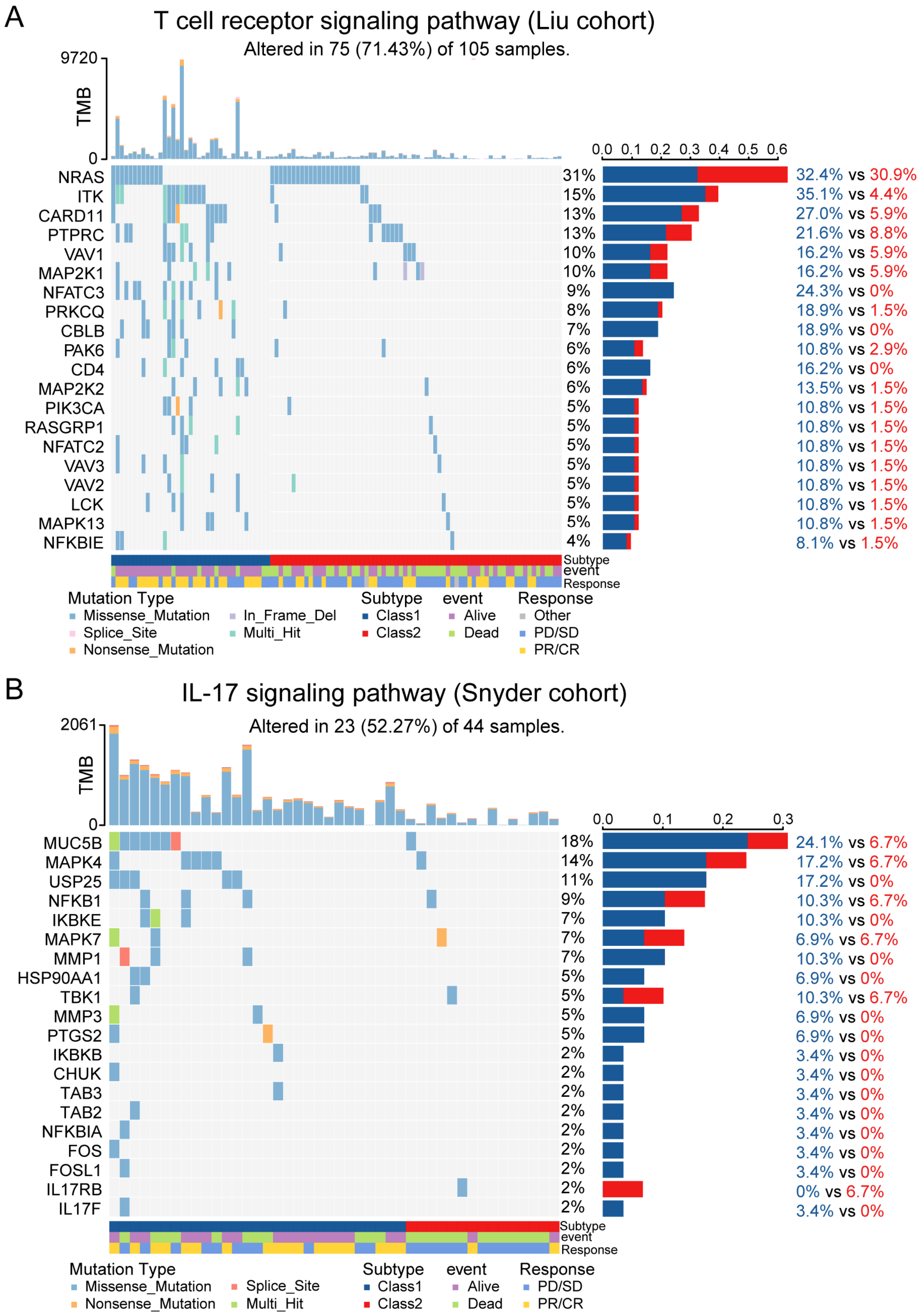

Supplementary Figure S5. Mutation analysis of genes involved in the characteristic pathways. (A) Waterfall plot of the top 20 genes with the highest mutation rates involved in the T cell receptor signaling pathway in the Liu cohort. (B) Waterfall plot of the top 20 genes with the highest mutation rates involved in the IL-17 signaling pathway in the Snyder cohort.

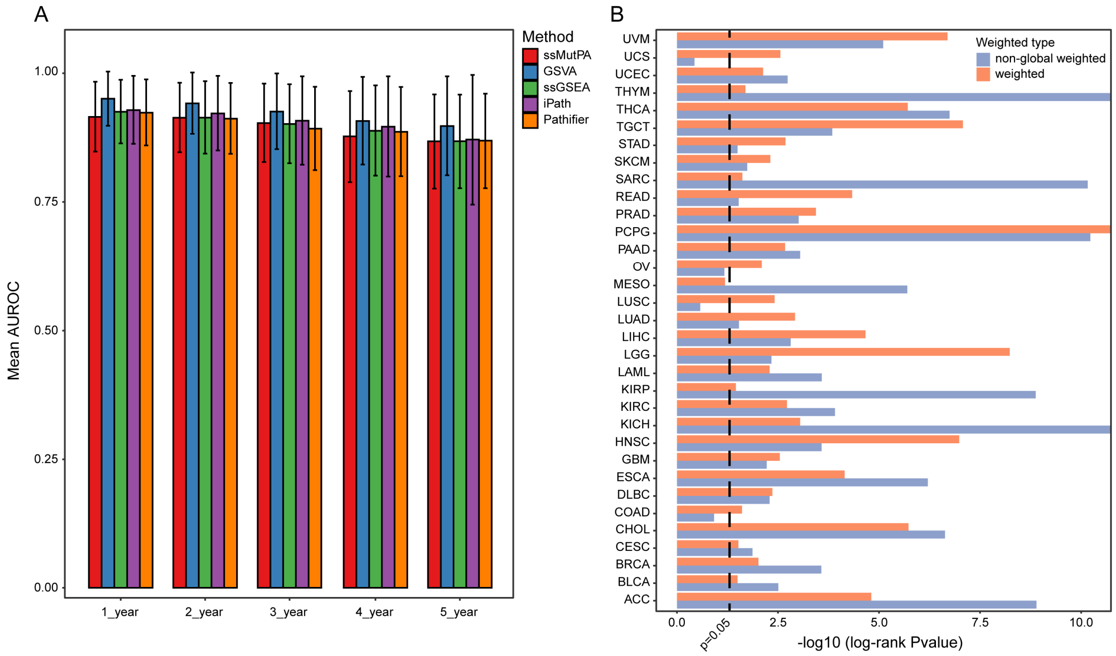

Supplementary Figure S6. Comparison of ssMutPA with other methods. **(A)** Histograms of mean AUROC for 1-5 year survival predictions across 14 cancer types using different methods. **(B)** Histograms of comparing the clustering performance (p-value of the log-rank test) of ssMutPA with non-global weighted ssMutPA across 33 cancer types.

Supplementary Table S1. The information of all the cohorts we used in this study.

(see Table S1-3_Supplementary Material.xlsx)

Supplementary Table S2. Detailed information on 33 cancer types in the TCGA database.

(see Table S1-3_Supplementary Material.xlsx)

Supplementary Table S3. The accession identifiers for all samples.

(see Table S1-3_Supplementary Material.xlsx)

Supplementary Table S4. Prognostically relevant pathway identified in glioma (cox p-value<0.05).

(see Table S4_Supplementary Material.xlsx)

Supplementary Table S5. Comparison of top 20 pathways identified by ssMutPA, ssGSEA, GSVA, iPath, and Pathifier in glioma.

Pathways	ssMutPA	ssGSEA	GSVA	iPath	Pathifier
Citrate cycle (TCA cycle)	√				
Glutathione metabolism	√	√	√		√
Glyoxylate and dicarboxylate metabolism	√				
Pentose phosphate pathway	√	√			
Lysine degradation	√				
Glycolysis / Gluconeogenesis	√			√	
Bacterial invasion of epithelial cells	√				
Platelet activation	√				
Phospholipase D signaling pathway	√				
Malaria	√				
Endocrine and other factor-regulated calcium reabsorption	√				
Rap1 signaling pathway	√				
Olfactory transduction	√				
Focal adhesion	√				√
Vascular smooth muscle contraction	√				√
Yersinia infection	√				
Homologous recombination	√				
ErbB signaling pathway	√				
Regulation of actin cytoskeleton	√				√
Leukocyte transendothelial migration	√				
