# Supplementary material for: ssMutPA: single-sample mutation-based pathway analysis approach for cancer precision medicine
Source: Gigascience. 2024 Dec 20;13:giae105. doi: 10.1093/gigascience/giae105 (PMC11659979; doi:10.1093/gigascience/giae105)
Supplement: giae105_Response_to_Reviewer_Comments_Original_Submission [file giae105_response_to_reviewer_comments_original_submission.pdf]

Author's Response To Reviewer Comments

Dear Prof. Hongling Zhou and reviewers:

Thank you for your letter and the valuable comments and suggestions provided by the reviewers on our manuscript entitled "ssMutPA: Single-sample Mutation-based Pathway Analysis approach for cancer precision medicine" (Manuscript ID: GIGA-D-24-00212). These comments are highly invaluable in revising and improving our paper and provide significant guidance for our research. We have carefully studied the comments and made corrections which we hope meet with approval. The revised sections are marked in RED in the paper. If you or the reviewers have any other suggestions, we are willing to revise them until you are satisfied. The main corrections in the paper and the response to the reviewers' comments are as follows:

Reply to Editor
Please register any new software application in the bio.tools and SciCrunch.org databases to receive RRID (Research Resource Identification Initiative ID) and biotoolsID identifiers, and include these in your manuscript. Computational workflows should be registered in workflowhub.eu and the DOIs cited in the relevant places in the manuscript. These will facilitate tracking, reproducibility and re-use of your tool.

Please ensure you describe additional experiments that were carried out and include a detailed rebuttal of any criticisms or requested revisions that you disagreed with. Please also ensure that your revised manuscript conforms to the journal style, which can be found in the Instructions for Authors on the journal homepage. If the data and code has been modified in the revision process please be sure to update the public versions of this too.

Response: Thank you very much for your editorial efforts regarding our manuscript and for inviting us to submit a revised version of our manuscript. In response to your request, we have registered our software on the bio.tools and SciCrunch.org database and added the RRID and biotoolsID identifiers to the section "Availability of Source Code and Requirements" in the main manuscripts. We have provided detailed responses to all the reviewers' comments. We can confirm that modifications have been made to the main manuscript and supplementary data, and additional experimental results have been included as required. Lastly, we have adjusted the overall style of the manuscript to ensure compliance with the journal's "Author Guidelines" section. We believe that the manuscript has been greatly strengthened by the critique of the reviewers and hope that both you and the reviewers will now find the paper suitable for publication.

Reply to Reviewer #1
He et al. proposed a Single-sample pathway enrichment analysis method (ssMutPA) using patient-specific somatic mutation data. This method ranks the patient's genes based on mutation data and PPI, utilizing a Local weighted strategy and a Global propagation-based weighted strategy. Subsequently, pathway enrichment is used to obtain a Single-sample pathway activity score. Experiments on the TCGA dataset confirmed that the Single-sample pathway activity score can cluster cancer patients into subtypes with different prognosis risks, and melanoma patients in different subtypes show varying responses to immunotherapy. The manuscript is well-organized, the work is substantial, and the logic is clear. I believe this is a valuable contribution. I have the following questions:

Response: Thank you very much for your comments concerning our manuscript. These comments are valuable and insightful to improve our work. According to your comments, we have comprehensively revised the manuscript.

Point 1: To evaluate the effectiveness of the authors' method, they compared ssMutPA with non-weighted ssMutPA, demonstrating that ssMutPA has better prognostic stratification ability in most cancer datasets, confirming that the local weighted strategy increases the performance of ssMutPA. The method has two important components: the Local weighted strategy and the Global propagation-based weighted strategy. I believe the authors should also compare the performance of methods using the Global propagation-based

weighted strategy versus those not using it (e.g., obtaining important gene lists based on local scores, then using hypergeometric distribution tests or other strategies to get pathway activity scores and perform the same analysis). This would confirm the significant contributions of both parts to the overall results.

Response 1: Thank you for your invaluable comment. As you mentioned, in ssMutPA, we use network-based local weighted and global propagation strategies to calculate the single-sample mutation-based pathway enrichment score (ssMutPES), and the comparative analysis indicated that local weighted enhances the performance of ssMutPA. However, as you correctly emphasized, this method has two crucial components, and global propagation is equally important. It is necessary to compare the performance of the method using the global weighted strategy versus one without it. Following your suggestion, we removed the global weighted strategy and recalculated the ssMutPES using the hypergeometric distribution test. Subsequently, we applied the same procedure to the ssMutPES profiles to identify cancer subtypes. By comparing the prognostic differences between various cancer subtypes, we found that the performance (p-value of the log-rank test) of ssMutPA outperformed non-global weighted ssMutPA in more cancer types (Supplementary Figure S6B). We believe that this comparison not only strengthens the explanation of the results but also ensures a fair evaluation.
We have added the relevant content to the manuscript and included the results in the Supplementary files as Supplementary Figure S6B.

Page 18, section "Comparison of ssMutPA with non-weighted ssMutPA" in the main manuscript.
"To assess if the local weighted strategy increases the performance of ssMutPA, we compared the clustering results of ssMutPA with that of ssMutPA without local weight (expressed as non-local weighted ssMutPA) across 33 cancers in TCGA. Specifically, we applied non-local weighted ssMutPA to each cancer type and obtained the ssMutPES profiles of pathways. Subsequently, the same procedure was performed on the ssMutPES profiles to identify cancer subtypes. By comparing the prognostic difference among subtypes (Kaplan-Meier survival analysis), we found that the performance (p-value of the log-rank test) of ssMutPA outperformed non-local weighted ssMutPA in almost all cancer types (Figure 7C). Moreover, in addition to local weighted, we introduced a global propagation-based weighted strategy to assess the impact of mutated genes on the activity of other genes within the network. To evaluate whether the global propagation strategy enhanced the performance of ssMutPA, we compared the clustering results of ssMutPA with the ssMutPA without global weighted (expressed as non-global weighted ssMutPA) across 33 cancer types from the TCGA database. After excluding the global propagation-based weights, we recalculated ssMutPES using the hypergeometric distribution test. Following the same comparison process, we found that ssMutPA's performance (log-rank test p-values) still outperformed non-global weighted ssMutPA (Supplementary Figure S6B). These results demonstrated that the local and global weighted strategies were essential to ssMutPA, which increases its predicted efficacy."

Page 22, section "Additional Files" in the main manuscript.
"Supplementary Figure S6. Comparison of ssMutPA with other methods."

Page 27, section "Figures legends" in the main manuscript.
"Figure 7. (C) Comparing the clustering performance (p-value of the log-rank test) of ssMutPA with non-local weighted ssMutPA across 33 cancer types."

Page 8, Supplementary Figure S6 in Supplementary Materials.
"Supplementary Figure S6. Comparison of ssMutPA with other methods. (A) Histograms of mean AUROC for 1-5 year survival predictions across 14cancer types using different methods. (B) Histograms of comparing the clustering performance (p-value of the log-rank test) of ssMutPA with non-global weighted ssMutPA across 33 cancer types."

Point 2: In Figures 7a and 7b, the authors compared the performance of ssMutPA with Single-sample pathway enrichment analysis methods based on transcriptome data. The results indicate that ssMutPA is not significantly better than the transcriptome-based methods, and these figures do not clearly compare the performance of different methods. I suggest the authors add a data metric to more intuitively describe the performance of each method (e.g., comparing the ranking of each method's performance across all datasets, then calculating the mean rank or another reasonable metric to show the performance of different methods). Although this method may not be significantly better than others, I still believe it has

value. As the authors said, "the ssMutPA method uses gene mutation data to calculate the pathway activities (ssMutPES), distinct from other methods that use gene expression data. Therefore, the ssMutPA method may provide some new insight into inferring individualized pathway activity and complement the current methods focused on gene expression data." To validate this point, I suggest the authors choose one or more cancers for a case study to demonstrate that ssMutPES can indeed identify important pathways in cancer prognosis that traditional methods cannot.

Response 2: Thank you very much for pointing this out. In our study, to explain whether the ssMutPA approach could provide new biological insights, we compared it with other individualized transcriptome-based pathway activity analysis methods, including GSVA, ssGSEA, iPath, and Pathifier. The comparison results indicated that ssMutPA can effectively predict cancer patient prognosis and outperforms other methods in some cancers. However, as you mentioned, we did not clearly elaborate on the overall performance of these methods. In response to your guidance, to provide a more comprehensive comparison of the individualized pathway activity analysis methods, we conducted a summary analysis of the area under the time-dependent ROC curve from one to five years across 14 types of cancer (Supplementary Figure S6A). The results show that, except for GSVA, the one-to-five-year average AUROC for the ssMutPA method is comparable to other methods. Moreover, to demonstrate that ssMutPA can indeed identify critical prognostic pathways neglected by other transcriptome-based methods, we further applied ssGSEA, GSVA, iPath, and Pathifier to TCGA-Glioma (LGG and GBM) expression data to calculate single-sample pathway activity profiles and identify prognosis-related pathways through univariate COX proportional hazards regression analysis. By comparing the top 20 pathways identified by ssMutPA with the results of other methods, we found that ssMutPA uniquely identified 14 prognostic pathways (Supplementary Table S5). Interestingly, some of these pathways, such as the Citrate cycle (TCA cycle) [PMID:38483541] and Rap1 signaling pathway [PMID: 9380414], have been confirmed to be associated with glioma prognosis in previous studies. The above results revealed that the ssMutPA method focused on mutation data may provide some new insight into inferring individualized pathway activity and complement the current methods focused on gene expression data.
We have added the above content to the main manuscript and included the corresponding results in Supplementary Figure S6A and Supplementary Table S5.

Page 17, section “Comparison of ssMutPA with other individualized pathway activity analysis methods based on transcriptomic data” in the main manuscript.
“Additionally, we performed time-dependent Receiver Operating Characteristic (ROC) curve analysis for 1-5 years for each method's signature. The results showed that the values of area under the ROC curve (AUROC) of the ssMutPA signature exceeded 0.75 in almost all cancers, which were also comparable to the signatures of other methods (Figure 7B and Supplementary Figure S6A). Finally, we applied ssGSEA, GSVA, iPath and Pathifier to TCGA-Glioma (LGG and GBM) gene expression data to calculate single-sample pathway activity profiles and identify prognosis-related pathways through univariate COX proportional hazards regression analysis. Comparing these results with the top 20 pathways identified by ssMutPA, we found that ssMutPA uniquely identified 14 pathways associated with patient prognosis (Supplementary Table S5). Most of these pathways, such as the Citrate cycle (TCA cycle) [50] and Rap1 signaling pathway [51], have been confirmed to be associated with glioma prognosis in previous studies. These results illustrated that the signature of ssMutPA could effectively predict the prognosis of cancer patients. More importantly, the ssMutPA method uses gene mutation data to calculate the pathway activities (ssMutPES), distinct from other methods that use gene expression data. Therefore, the ssMutPA method may provide some new insight into inferring individualized pathway activity and complement the current methods focused on gene expression data.”

Page 22,section “Additional Files” in the main manuscript.
“Supplementary Table S5. Comparison of top 20 pathways identified by ssMutPA, ssGSEA, GSVA, iPath, and Pathifier in glioma.”

Page 8, Supplementary Figure S6 in Supplementary Materials.
“Supplementary Figure S6. Comparison of ssMutPA with other methods. (A) Mean AUROC for 1-5 year survival predictions across 14 cancer types using different methods. (B) Comparing the clustering performance (p-value of the log-rank test) of ssMutPA with non-global weighted ssMutPA.”

Point 3: During clustering, the authors first used Cox regression to filter pathways and then used the activity scores of these pathways to cluster cancers. Due to the Cox regression filtering process, I believe that under these circumstances, different clusters of cancer patients will definitely show significant differences in prognosis risks. For example, the authors could directly use Cox regression to select significant genes and then cluster cancer patients based on the expression values of these significant genes. I believe even in this case, patients in different cancer categories would show significant differences in survival risks. I find the approach of using Cox regression to filter pathways beforehand to be somewhat unreasonable. I am curious about how ssMutPA would perform without the preliminary Cox filtering.

Response 3: We sincerely appreciate your valuable suggestions. In recent years, we have noticed that many pathway analysis methods have been proposed, but the majority of these methods (such as ssGSEA, GSVA, iPath and Pathifier) focus exclusively on gene expression data, overlooking somatic mutation information. Therefore, the main aim of this study is to innovatively propose ssMutPA by integrating somatic mutation data with the protein-protein interaction network. ssMutPA calculates mutation-based pathway activity scores for each pathway in each sample through local weighted and global propagation strategies, which can be used for subsequent clinical applications and analyses. To demonstrate the clinical applicability of mutation-induced pathway activity score (ssMutPES) profiles, we performed clustering analysis to identify cancer subtypes.
As you mentioned, to perform clustering analysis, we first used Cox regression to filter pathways and then used the activity scores of these pathways to cluster cancer samples. This is because not all pathways play a role in cancer progress. Thus, the Cox regression to filter pathways could prevent pathways that are not related to prognosis from introducing bias into the clustering analysis and obtain more accurate clustering results which may help to perform precise treatment according to different subtypes. If we perform without the preliminary Cox filtering, it may not produce stable and reproducible clustering results, which will limit the clinical applicability of our method. The Cox regression analysis or differential expression analysis to select significant pathways was a commonly used strategy by the recent pathway activity analysis methods (such as GSVA, iPath and Pathifier) to demonstrate the effectiveness of identifying dysregulated pathways. Moreover, because of the high heterogeneity of cancer, the gene-level biomarkers were generally limited by the reproducibility and effectiveness. Biological pathways reflecting the key cellular mechanisms that dictate disease states, drug response and altered cellular function could help to identify more accurate biomarkers. Currently, an increasing number of single-sample pathway activity calculation methods and tools have been developed for identifying dysregulated pathways in complex diseases. However, almost all methods focus on gene expression data, overlooking the gene mutation data because of its sparseness and discreteness. In this study, ssMutPA was developed to infer individualized pathway activities by integrating somatic mutation data and PPI network topology. ssMutPA may complement the existing methods focused on gene expression data and provide some new insights into cancer precision medicine.
We have added a detailed explanation to the "Discussion" section in the main manuscript.

Page19, section "Discussions" in the main manuscript.
"As the high heterogeneity of cancer, the gene-level biomarkers were generally limited by the instability. The pathways reflecting the key biological processes and cellular functions could help to identify more effective and reproducible biomarkers. Therefore, an increasing number of single-sample pathway activity calculation methods and tools are being developed for identifying dysregulated pathways in complex diseases [14-17]. However, almost all methods focus on gene expression data, overlooking the gene mutation data because of its sparseness and discreteness. In this study, ssMutPA was developed to infer individualized pathway activities by integrating somatic mutation data and PPI network topology. To demonstrate the effectiveness of ssMutPA, we applied it to 33 cancer types from the TCGA database. Based on the mutation-induced pathway activity (ssMutPES) profiles, the patients could be clustered into different subtypes with significantly different prognoses in each cancer type. When comparing the ssMutPA approach with other individualized pathway activity analysis methods, including GSVA, ssGSEA, iPath, and Pathifier, we found that the prognosis prediction power of ssMutPA-based signature was superior to other methods. This indicated that the mutation-based individualized pathway analysis may complement the existing methods focused on gene expression data and provide some new insights into cancer precision medicine."

Reply to Reviewer #2
This article is data mining, and the author is invited to focus on its innovations. In addition, can this article

be further experimentally verified? Finally, the R version is older, please describe why you use it.

Response: We appreciate your valuable feedback on our manuscript. Currently, numerous single-sample pathway enrichment analysis methods have been proposed, but most of them mainly focus on gene expression data. In clinical practice, indeed, mutation data is more commonly used than other omics analyses. Therefore, to complement existing pathway analysis methods, we innovatively proposed a single-sample mutation-based pathway analysis method (ssMutPA). This method integrates somatic mutation data with the PPI network and calculates single-sample mutation-based pathway enrichment score (ssMutPES) using the local weighted and global propagation strategies. Specifically, the local weighted strategy considers the mutation frequency of the neighboring nodes of the seed nodes, while the global weighted quantifies the impact of the mutations on other genes in the network through an iterative propagation algorithm. By focusing on mutation data, ssMutPA facilitates clinical application, addresses the limitations of existing methods, advances precision medicine, and provides new insights for individualized treatment.
Moreover, to verify the effectiveness of this method, we first test whether ssMutPA could effectively identify aberrant pathways associated with clinical prognosis. We applied it to 33 cancer types from the TCGA to calculate the ssMutPES profiles and used univariate COX proportional hazards regression for pathway filtering. The results showed that several important prognosis-related pathways were identified across different cancers, including Mismatch repair, T cell receptor signaling pathway, MAPK signaling pathway, etc. These pathways have been reported by previous studies to be associated with the occurrence and progression of cancers. Subsequently, we constructed the patient-patient similarity matrix based on the prognostic pathways and utilized spectral clustering algorithms to classify samples into different subtypes for each cancer type. Comparative analysis revealed significant prognostic differences among subtypes across all cancer types except MESO. These findings suggested that ssMutPA can be effectively and widely employed across various cancers, enabling downstream analysis. Additionally, we applied this method to somatic mutation data from two independent glioma validation cohorts in addition to TCGA-Glioma. By comparing the prognostic pathways identified in the three datasets, we observed a high degree of overlap. Specifically, the prognostic pathways identified in the Varn cohort and Wang cohort overlapped with those of TCGA-Glioma by 90.48% and 80.65%, respectively. These results not only indicated that ssMutPA is suitable for data from diverse sources but also highlighted the robustness of our method.
Although this study did not conduct wet experiments, we validated the broad applicability and robustness of ssMutPA through the experiments in silico and published literature, and further emphasizing the method's innovation and effectiveness. For these reasons, we chose to submit our manuscript as a "Technical Note". The requirements for this type of article are described as follows: "Technical Notes should present an open-source software tool or an experimental or computational method, test or procedure for the analysis or handling of large-scale data. The tool or method described may be new, or an update or adaptation of an existing one." Therefore, ssMutPA introduces a novel single-sample pathway enrichment method based on somatic mutation data, addressing the limitations of existing methods, and which meets the requirements of the journal.
Regarding the R version used, we would like to clarify that the ssMutPA package was developed and tested using R version 4.3.0. However, to ensure broader accessibility and usability for users working with different versions of R, we set the minimum required version to 4.0.0. This allows users with older versions of R to still effectively use the package.
Thank you again for your comments on our work. We have added and strengthened the description of the innovation and results of the ssMutPA in the main manuscript. If you have any other suggestions, we are willing to revise them until you are satisfied.

Page 14-15, section "Application of the ssMutPA approach in glioma"
"Finally, to assess the generalizability and robustness of the ssMutPA method, we collected two independent glioma datasets (Varn et al. cohort and Wang et al. cohort) from cBioPortal. ssMutPA was respectively applied to these two datasets to calculate ssMutPES profiles, followed by the same process to identify significant prognosis-related pathways. Through comparing the pathways with the results from the TCGA-Glioma cohort, we observed 90.48% and 80.65% of the significant pathways identified in the Varn et al. cohort and Wang et al. cohort overlapped with those of in TCGA-Glioma cohort (Supplementary Figure S4E). Moreover, we also performed the robustness analysis to test the influence of the network structure. Specifically, we randomly removed 5%, 10%, 15%, and 20% of the edges from the original network respectively and recalculated the ssMutPES profiles for each removal, and then identified the prognosis-

related pathways based on the ssMutPES profiles. We found that the percentage of overlapped prognostic related pathways to original significant pathways remained above 80%, even after the removal of up to 20% of the edges (Supplementary Figure S4F)."

Page 17, section "Comparison of ssMutPA with other individualized pathway activity analysis methods based on transcriptomic data" in the main manuscript.
"Finally, we applied ssGSEA, GSVA, iPath and Pathifier to TCGA-Glioma (LGG and GBM) gene expression data to calculate single-sample pathway activity profiles and identify prognosis-related pathways through univariate COX proportional hazards regression analysis. Comparing these results with the top 20 pathways identified by ssMutPA, we found that ssMutPA uniquely identified 14 pathways associated with patient prognosis (Supplementary Table S5). Most of these pathways, such as the Citrate cycle (TCA cycle) [50] and Rap1 signaling pathway [51], have been confirmed to be associated with glioma prognosis in previous studies."

Page 18, section "Comparison of ssMutPA with non-weighted ssMutPA" in the main manuscript.
"Moreover, in addition to local weighted, we introduced a global propagation-based weighted strategy to assess the impact of mutated genes on the activity of other genes within the network. To evaluate whether the global propagation strategy enhanced the performance of ssMutPA, we compared the clustering results of ssMutPA with the ssMutPA without global weighted (expressed as non-global weighted ssMutPA) across 33 cancer types from the TCGA database. After excluding the global propagation-based weights, we recalculated ssMutPES using the hypergeometric distribution test. Following the same comparison process, we found that ssMutPA's performance (log-rank test p-values) still outperformed non-global weighted ssMutPA (Supplementary Figure S6B). These results demonstrated that the local and global weighted strategies were essential to ssMutPA, which increases its predicted efficacy."

Page 19, section "Discussion" in the main manuscript.
"As the high heterogeneity of cancer, the gene-level biomarkers were generally limited by the instability. The pathways reflecting the key biological processes and cellular functions could help to identify more effective and reproducible biomarkers."

Reply to Reviewer #3
The authors present ssMutPA, a novel computational method for inferring individualized pathway activities from somatic mutation data using protein-protein interaction (PPI) networks and KEGG pathways. The authors apply ssMutPA to analyze tumor samples and predict immunotherapy responses in melanoma cohorts. The idea focusing on the somatic mutations is interesting, but there are several issues to be clarified.

Response: Thank you very much for your comments on our manuscript. These suggestions provided valuable guidance and assistance in improving the quality of our work. We have carefully considered each of your comments and made revisions to the manuscript.

Point 1: The authors used PPI networks for the weight calculation. Please explain more clearly why PPI networks were chosen for weighting. It might be possible to use the KEGG pathways even at the weighting schemes. Or mutation frequencies can be used for weights.

Response 1: I would like to express my gratitude for your comment. While KEGG pathways are often used to construct network in some cases, we chose to use PPI networks for weighting in this study for several reasons. First, we integrated the PPI network from 12 sources and selected high-confidence links. Compared to KEGG pathways, which include 323 pathways with 5954 genes and 61632 interactions, the PPI network encompasses a broader range of human genes (12,436 genes) and more extensive protein interactions (83,020 edges). Second, KEGG pathways are based on existing biological knowledge, which introduces some bias and may not be suitable for comprehensive global network analysis. Therefore, compared to using KEGG pathways to construct the network, the PPI network is more suitable for applying local weighted and global propagation strategies.
Additionally, ssMutPA was developed as a novel computational method for inferring individualized pathway activities from somatic mutation data using protein-protein interaction (PPI) networks and KEGG pathways.

However, mutation frequencies consider the extent of mutation in a cancer cohort and could not reflect individualized impact. Thus, ssMutPA uses local and global weighted strategies to evaluate the effects of genes from mutations according to the network topology and then calculates a single-sample mutation-based pathway enrichment score (ssMutPES) to quantify the accumulated effect of mutations of each pathway.
We have added a detailed description of our choice to use PPI networks in the manuscript.

Page 6, section "Data collection and processing" in the manuscript.
"The human-specific PPI network was obtained from 12 sources collected by previous researchers [35, 36]. To obtain high-confidence links, we further filtered PPIs from more than two sources. This measure for filtering PPIs has been used in previous studies [13]. Finally, the largest connected sub-network containing 12436 nodes and 83020 edges was extracted using the "igraph" package. Compared to directly using KEGG pathways for network construction (323 pathways with 5954 genes), the PPI network encompasses a more comprehensive human genes."

Point 2: The authors compare ssMutPA to a non-weighted version, demonstrating modest improvement with local weighting. However, the authors do not show the results without global weighting (i.e., without the Random Walk with Restart algorithm). Please clarify the effects on the global weighting with the experimental results.

Response 2: Thank you for your invaluable comment. In ssMutPA, we use network-based local weighted and global propagation strategies to calculate the single-sample mutation-based pathway enrichment score (ssMutPES), and the comparative analysis demonstrated that local weighted enhances the performance of ssMutPA. However, as you correctly emphasized, we did not include a comparison with the method that does not use global propagation. It is necessary to compare the performance of the method using a global weighted strategy versus one without it. Following your suggestion, we removed the global weighted strategy and calculated the ssMutPES using the hypergeometric distribution test. Subsequently, we applied the same procedure to the ssMutPES profiles to identify cancer subtypes. By comparing the prognostic differences between various cancer subtypes, we found that ssMutPA still outperforms ssMutPA without global propagation (non-global weighted ssMutPA) (Figure S6B). Specifically, ssMutPA could identify significant prognostic difference subtypes in 32 out of 33 cancer types; however non-global weighted ssMutPA did not identify significant prognostic difference subtypes in four cancer types. These results demonstrated that the local and global weighted strategies were essential to ssMutPA, which increases its predicted efficacy.
We believe that this comparison not only strengthens the explanation of the results but also ensures fairness in the evaluation. We have added the relevant content to the manuscript and included the results in the Supplementary files as Supplementary Figure S6B.

Page 18, section "Comparison of ssMutPA with non-weighted ssMutPA" in the main manuscript.
"To assess if the local weighted strategy increases the performance of ssMutPA, we compared the clustering results of ssMutPA with that of ssMutPA without local weight (expressed as non-local weighted ssMutPA) across 33 cancers in TCGA. Specifically, we applied non-local weighted ssMutPA to each cancer type and obtained the ssMutPES profiles of pathways. Subsequently, the same procedure was performed on the ssMutPES profiles to identify cancer subtypes. By comparing the prognostic difference among subtypes (Kaplan-Meier survival analysis), we found that the performance (p-value of the log-rank test) of ssMutPA outperformed non-local weighted ssMutPA in almost all cancer types (Figure 7C). Moreover, in addition to local weighted, we introduced a global propagation-based weighted strategy to assess the impact of mutated genes on the activity of other genes within the network. To evaluate whether the global propagation strategy enhanced the performance of ssMutPA, we compared the clustering results of ssMutPA with the ssMutPA without global weighted (expressed as non-global weighted ssMutPA) across 33 cancer types from the TCGA database. After excluding the global propagation-based weights, we recalculated ssMutPES using the hypergeometric distribution test. Following the same comparison process, we found that ssMutPA's performance (log-rank test p-values) still outperformed non-global weighted ssMutPA (Supplementary Figure S6B). These results demonstrated that the local and global weighted strategies were essential to ssMutPA, which increases its predicted efficacy."

Page 22, section "Additional Files" in the main manuscript.

“Supplementary Figure S6. Comparison of ssMutPA with other methods. ”

Page 27, section “Figures legends” in the main manuscript.
“Figure 7. (C) Comparing the clustering performance (p-value of the log-rank test) of ssMutPA with non-local weighted ssMutPA across 33 cancer types.”

Page 8, Supplementary Figure S6 in Supplementary Materials.
“Supplementary Figure S6. Comparison of ssMutPA with other methods. (A) Histograms of mean AUROC for 1-5 year survival predictions across 14 cancer types using different methods. (B) Histograms of comparing the clustering performance (p-value of the log-rank test) of ssMutPA with non-global weighted ssMutPA across 33 cancer types.”

Point 3: The authors show the experimental results on TCGA datasets. For more generalizability and robustness of their proposed method, it is necessary to validate using independent and external datasets not derived from TCGA.

Response 3: Thank you for your invaluable comment. According to your suggestion, we downloaded two independent glioma datasets, Varn et al. cohort and Wang et al. cohort from cBioPortal to further validate the method. We applied ssMutPA to these datasets to calculate single-sample mutation-based pathway enrichment score profiles, followed by the same process to identify prognosis-related pathways. When comparing the pathways with the results from the TCGA-Glioma cohort, we observed 90.48% and 80.65% of the significant pathways identified in the Varn et al. cohort and Wang et al. cohort overlapped with those of in TCGA-Glioma cohort (Supplementary Figure S4E). The result not only indicates the generalizability of ssMutPA across different data sources but also confirms its ability to identify consistent prognostic pathways for the same cancer type, further highlighting the robustness of the method.
We have included the relevant analyses into the main manuscript and presented the corresponding results and data in Supplementary Figure S4E, Supplementary Table S1 and Supplementary Table S3.

Page 5, section “Data collection and processing” in the main manuscript.
“In addition, we obtained two independent glioma datasets (Varn et al. cohort and Wang et al. cohort) from the cBioPortal (https://www.cbioportal.org/) to validate the generalizability and robustness of the ssMutPA method .”

Page 14-15, section “Application of the ssMutPA approach in glioma” in the main manuscript.
“Finally, to assess the generalizability and robustness of the ssMutPA method, we collected two independent glioma datasets (Varn et al. cohort and Wang et al. cohort) from cBioPortal. ssMutPA was respectively applied to these two datasets to calculate ssMutPES profiles, followed by the same process to identify significant prognosis-related pathways. Through comparing the pathways with the results from the TCGA-Glioma cohort, we observed 90.48% and 80.65% of the significant pathways identified in the Varn et al. cohort and Wang et al. cohort overlapped with those of in TCGA-Glioma cohort (Supplementary Figure S4E). Moreover, we also performed the robustness analysis to test the influence of the network structure. Specifically, we randomly removed 5%, 10%, 15%, and 20% of the edges from the original network respectively and recalculated the ssMutPES profiles for each removal, and then identified the prognosis-related pathways based on the ssMutPES profiles. We found that the percentage of overlapped prognostic related pathways to original significant pathways remained above 80%, even after the removal of up to 20% of the edges (Supplementary Figure S4F).”

Page 21,section “Additional Files” in the main manuscript.
“Supplementary Figure S4. Analysis of immune features between glioma subtypes and robustness assessment of the ssMutPA method.”

Page 6, Supplementary Figure S4 in Supplementary Materials.
“Supplementary Figure S4. Analysis of immune features between glioma subtypes and robustness assessment of the ssMutPA method. (A) Box plot of the abundance of significant immune cells between glioma subtypes. The p-value at the top were calculated by the Wilcoxon rank-sum test. (B-D) Box plots of stromal score, immune score, and tumor purity between glioma subtypes. (E) Venn diagram of the number of overlapped significant pathways identified in the Varn et al. cohort and Wang et al. cohort with those of

in TCGA-Glioma cohort. (F) Histograms of the overlap number between prognostic pathways identified in TCGA-Glioma after removing different proportions of edges from the network and the original prognostic pathways.”

Point 4: The reliability of the proposed method may depend heavily on the quality and completeness of the PPI network used. The authors should discuss this limitation and its potential impacts.

Response 4: Thank you very much for your comments. In this paper, although we used the PPI network that integrates high-confidence links from 12 sources, limitations in current research on gene relationships still result in some loss of interactions. Based on your suggestion, we further discussed the impacts of PPI network integrity on the ssMutPA method and conducted robustness evaluations. Specifically, we randomly removed 5%, 10%, 15%, and 20% of the edges from the original network and recalculated the ssMutPES profiles for each removal, and then identified the prognosis-related pathways. We found that the percentage of overlapped prognosis-related pathways to original significant pathways remained above 80%, even after the removal of up to 20% of the edges (Supplementary Figure S4F). Although we demonstrated the robustness of the ssMutPA method to the network structure, the ssMutPA method may also limited by the incomplete PPI network. With the PPI network constantly updated, the applicability of the ssMutPA method will be further enhanced.
We have added relevant analyses on the quality and completeness of the PPI network as well as discussed the limitations of the PPI network on our method. The results of the robustness analysis of the ssMutPA method on the PPI network structure are shown in Supplementary Figure S4F.

Page 15, section “Application of the ssMutPA approach in glioma” in the main manuscript.
“Moreover, we also performed the robustness analysis to test the influence of the network structure. Specifically, we randomly removed 5%, 10%, 15%, and 20% of the edges from the original network and recalculated the ssMutPES profiles for each removal, and then identified the prognosis-related pathways based on the ssMutPES profiles. We found that the percentage of overlapped prognostic related pathways to original significant pathways remained above 80%, even after the removal of up to 20% of the edges (Supplementary Figure S4F).”

Page 20, section “Discussion” in the main manuscript.
“Although we demonstrated the robustness of the ssMutPA method to the network structure, the method may also limited by the incomplete PPI network. With the PPI network constantly updated, the applicability of the ssMutPA method will be further enhanced.”

Page 6, Supplementary Figure S4 in Supplementary Materials.
“Supplementary Figure S4. Analysis of immune features between glioma subtypes and robustness assessment of the ssMutPA method. (A) Box plot of the abundance of significant immune cells between glioma subtypes. The p-value at the top were calculated by the Wilcoxon rank-sum test. (B-D) Box plots of stromal score, immune score, and tumor purity between glioma subtypes. (E) Venn diagram of the number of overlapped significant pathways identified in the Varn et al. cohort and Wang et al. cohort with those of in TCGA-Glioma cohort. (F) Histograms of the overlap number between prognostic pathways identified in TCGA-Glioma after removing different proportions of edges from the network and the original prognostic pathways.”
