# Supplementary material for: ssMutPA: single-sample mutation-based pathway analysis approach for cancer precision medicine
Source: Gigascience. 2024 Dec 20;13:giae105. doi: 10.1093/gigascience/giae105 (PMC11659979; doi:10.1093/gigascience/giae105)
Supplement: giae105_Reviewer_1_Report_Original_Submission — Xionghui Zhou -- 6/26/2024 Reviewed [file giae105_reviewer_1_report_original_submission.pdf]

Reviewer Report

Title: ssMutPA: Single-sample Mutation-based Pathway Analysis approach for cancer precision medicine

Version: Original Submission **Date:** 6/26/2024

Reviewer name: Xionghui Zhou

Reviewer Comments to Author:

He et al. proposed a Single-sample pathway enrichment analysis method (ssMutPA) using patient-specific somatic mutation data. This method ranks the patient's genes based on mutation data and PPI, utilizing a Local weighted strategy and a Global propagation-based weighted strategy. Subsequently, pathway enrichment is used to obtain a Single-sample pathway activity score. Experiments on the TCGA dataset confirmed that the Single-sample pathway activity score can cluster cancer patients into subtypes with different prognosis risks, and melanoma patients in different subtypes show varying responses to immunotherapy. The manuscript is well-organized, the work is substantial, and the logic is clear. I believe this is a valuable contribution. I have the following questions:(1)To evaluate the effectiveness of the authors' method, they compared ssMutPA with non-weighted ssMutPA, demonstrating that ssMutPA has better prognostic stratification ability in most cancer datasets, confirming that the local weighted strategy increases the performance of ssMutPA. The method has two important components: the Local weighted strategy and the Global propagation-based weighted strategy. I believe the authors should also compare the performance of methods using the Global propagation-based weighted strategy versus those not using it (e.g., obtaining important gene lists based on local scores, then using hypergeometric distribution tests or other strategies to get pathway activity scores and perform the same analysis). This would confirm the significant contributions of both parts to the overall results.(2)In Figures 7a and 7b, the authors compared the performance of ssMutPA with Single-sample pathway enrichment analysis methods based on transcriptome data. The results indicate that ssMutPA is not significantly better than the transcriptome-based methods, and these figures do not clearly compare the performance of different methods. I suggest the authors add a data metric to more intuitively describe the performance of each method (e.g., comparing the ranking of each method's performance across all datasets, then calculating the mean rank or another reasonable metric to show the performance of different methods). Although this method may not be significantly better than others, I still believe it has value. As the authors said, "the ssMutPA method uses gene mutation data to calculate the pathway activities (ssMutPES), distinct from other methods that use gene expression data. Therefore, the ssMutPA method may provide some new insight into inferring individualized pathway activity and complement the current methods focused on gene expression data." To validate this point, I suggest the authors choose one or more cancers for a case study to demonstrate that ssMutPES can indeed identify important pathways in cancer prognosis that traditional methods cannot.(3)During clustering, the authors first used Cox regression to filter pathways and then used the activity scores of these pathways to cluster cancers. Due to the Cox regression filtering process, I believe that under these circumstances, different clusters of cancer patients will definitely show significant

differences in prognosis risks. For example, the authors could directly use Cox regression to select significant genes and then cluster cancer patients based on the expression values of these significant genes. I believe even in this case, patients in different cancer categories would show significant differences in survival risks. I find the approach of using Cox regression to filter pathways beforehand to be somewhat unreasonable. I am curious about how ssMutPA would perform without the preliminary Cox filtering.

Level of Interest

Please indicate how interesting you found the manuscript: Choose an item.

Quality of Written English

Please indicate the quality of language in the manuscript: Choose an item.

Declaration of Competing Interests

Please complete a declaration of competing interests, considering the following questions:

- Have you in the past five years received reimbursements, fees, funding, or salary from an organisation that may in any way gain or lose financially from the publication of this manuscript, either now or in the future?
- Do you hold any stocks or shares in an organisation that may in any way gain or lose financially from the publication of this manuscript, either now or in the future?
- Do you hold or are you currently applying for any patents relating to the content of the manuscript?
- Have you received reimbursements, fees, funding, or salary from an organization that holds or has applied for patents relating to the content of the manuscript?
- Do you have any other financial competing interests?
- Do you have any non-financial competing interests in relation to this paper?

If you can answer no to all of the above, write 'I declare that I have no competing interests' below. If your reply is yes to any, please give details below.

I declare that I have no competing interests.

I agree to the open peer review policy of the journal. I understand that my name will be included on my report to the authors and, if the manuscript is accepted for publication, my named report including any attachments I upload will be posted on the website along with the authors' responses. I agree for my report to be made available under an Open Access Creative Commons CC-BY license (http://creativecommons.org/licenses/by/4.0/). I understand that any comments which I do not wish to

be included in my named report can be included as confidential comments to the editors, which will not be published.

Choose an item.

To further support our reviewers, we have joined with Publons, where you can gain additional credit to further highlight your hard work (see: https://publons.com/journal/530/gigascience). On publication of this paper, your review will be automatically added to Publons, you can then choose whether or not to claim your Publons credit. I understand this statement.

Yes Choose an item.
